# Lifestyle Intervention for Sustained Remission of Metabolic Syndrome: A Randomized Clinical Trial

**DOI:** 10.1001/jamainternmed.2025.5900

**Published:** 2025-11-09

**Authors:** Lynda H. Powell, Jannette Berkley-Patton, Betty M. Drees, Kelly Karavolos, Barbara Lohse, Kevin S. Masters, Jacinda M. Nicklas, Steven K. Rothschild, Chen Yeh, Laura J. Zimmermann, Sumihiro Suzuki

**Affiliations:** 1 Department of Family and Preventive Medicine, Rush University Medical Center, Chicago, Illinois; 2 Department of Biomedical and Health Informatics, University of Missouri, Kansas City; 3 Wegmans School of Health and Nutrition, Rochester Institute of Technology, Rochester, New York; 4 Anschutz Medical Campus, University of Colorado, Denver; 5 School of Medicine, University of Colorado, Denver; 6 Department of Internal Medicine, Rush University Medical Center, Chicago, Illinois

## Abstract

**Importance:**

Prevalence of metabolic syndrome (MetS) defined as 3 or more cardiometabolic risk factors has grown to 43% among US adults. Lifestyle interventions produce benefits, but with limited sustainability.

**Objective:**

To determine whether a 6-month habit-based lifestyle program adds benefit to education and activity monitoring for sustained MetS remission at 24 months.

**Design, Setting, and Participants:**

The Enhancing Lifestyles in Metabolic Syndrome (ELM) study was a single-blind, individually randomized clinical trial of participants with MetS recruited from July 2019 to January 2022 at 5 locations in the US and followed up for 24 months. Eligible participants were motivated for lifestyle change and were contacted using electronic medical records and social or mass media; those who did not meet medical or logistical criteria were excluded. In all, 14 817 adults were screened during a 2.5 year period. Data were analyzed from March 2024 to May 2025.

**Interventions:**

All participants received education and an activity monitor. Those in the intervention group also received 19 small group in-person meetings during 6 months, which sought to establish the following habits as part of their daily routine: vegetables at meals, brisk walks, sensory awareness, and emotion regulation. Repetition of these habits was encouraged by attention to participants’ experience of immediate benefits and peer support. The comparator group received 24 evidence-based educational mailings monthly during the 24 months.

**Main Outcome and Measures:**

MetS remission at 24 months determined by blinded laboratory evaluation and clinical examination. Secondary outcomes were cardiometabolic and lifestyle risk factors at 6 and 24 months.

**Results:**

The analysis included 618 participants (mean [SD] age, 55.5 [11.0] years; 468 female [74.7%] and 150 male [24.3%] individuals), of whom 306 (49.5%) were randomized to the intervention and 312 (50.5%) to the comparator. The 24-month follow-up period was completed by 517 participants (83.7%). Obesity, measured by body mass index, was present in 513 participants (83%). At 6 months, the proportion achieving MetS remission was 24.8% in intervention group and 17.9% in comparator (adjusted odds ratio, 1.64; 95% CI, 1.07-2.53; *P* = .03), supported by improvements in waist circumference, triglycerides, fasting glucose, body mass index, weight, hemoglobin A_1c_, MetS severity, perceived stress, vegetable intake, moderate-intensity physical activity, daily steps, sensory awareness, and the habits of daily brisk walks and vegetables at meals. At 24 months, the proportion achieving sustained MetS remission was 27.8% (85 of 306 participants) in intervention group and 21.2% (66 of 312) in the comparator (adjusted odds ratio, 1.46; 95% CI, 1.01-2.14; *P* < .05), supported by sustained improvement in fasting glucose, vegetable intake, daily steps, sensory awareness, and the habit of daily brisk walks.

**Conclusions and Relevance:**

This randomized clinical trial found that sustained MetS remission after treatment may be possible by promoting simple habits through a behavior-based lifestyle program focused on immediate benefits.

**Trial Registration:**

ClinicalTrials.gov Identifier: NCT04036006

## Key Points

**Question:**

Does a 6-month habit-based group lifestyle intervention added to routine education and activity monitoring improve the proportion of patients with metabolic syndrome (MetS) who sustain remission at 24 months?

**Findings:**

This multisite randomized clinical trial of 618 participants with MetS found a significant difference in sustained MetS remission at 24 months in the lifestyle intervention group (28%) compared with the comparator group (21%).

**Meaning:**

Addition of a 6-month habit-forming lifestyle program to routine education and activity monitoring shows that sustained MetS remission after treatment may be possible by promoting simple habits and focusing on immediate benefits.

## Teaser

This multisite randomized clinical trial evaluates a 6-month habit-based lifestyle program added to education and activity monitoring in patients with metabolic syndrome for sustained remission at 24 months.

## Introduction

Metabolic syndrome (MetS) is diagnosed by the co-occurrence of 3 or more of 5 cardiometabolic risk factors: abdominal obesity, hypertension, elevated fasting glucose, elevated triglycerides, and low high-density lipoprotein (HDL) cholesterol.^1^ In the US, the prevalence of MetS in adults was 25.3% from 1988 to 1994 and increased 43.3% from 2017 to 2018.^2,3,4^ Additionally, MetS has grown among subgroups such as young adults, Asian American individuals, and the college educated.^3,4^ MetS is a multisystem risk factor for chronic and infectious diseases^5,6,7,8^ and is associated with elevated health care costs.^7,9,10^ Poor diet quality and physical inactivity are strong modifiable risk factors.^7,11,12,13^ Three high-quality trials with large, diverse populations of participants with MetS evaluated the impact of diet, with or without physical activity, on MetS remission for 24 months or longer.^14,15,16^ They achieved, on average, 22% MetS remission using lifestyle treatments that continued at various levels of intensity over the duration of the follow-up. No trial has demonstrated sustained remission of MetS after treatment has ended.

The Enhanced Lifestyles for Metabolic Syndrome (ELM) multisite efficacy trial tested the hypothesis that motivated participants with MetS receiving a 6-month habit-based lifestyle intervention in addition to education and activity monitoring would achieve a higher proportion of sustained 24-month MetS remission than those receiving education and activity monitoring alone. Secondary objectives compared cardiometabolic and lifestyle risk factors between groups at 6 and 24 months.

## Methods

A central institutional review board at Rush Medical Center approved the trial protocol (available in Supplement 1) and all amendments. Details of design, eligibility, recruitment, interventions, assessments, and baseline characteristics have been published elsewhere.^17^ Trial organization included an independent data coordinating center and a data and safety monitoring board. Written informed consent was obtained (eMethods 1 and 2 in Supplement 2), and the study followed the Consolidated Standards of Reporting Trials (CONSORT) reporting guideline^18^ and the Standard Protocol Items: Recommendations for Interventional Trials (SPIRIT) reporting guideline.^19^

### Design and Participants

This was a single-blind, individually randomized behavioral efficacy clinical trial of participants with MetS recruited from 5 diverse geographic regions: Rochester, New York; central and northeast Pennsylvania; Chicago, Illinois; Kansas City, Missouri; and Denver, Colorado. Recruitment was conducted from July 2019 through January 2022, with the trial ending in April 2024 as planned. To minimize bias from a single-blind design, blinding was extended to all operations using neutral names for trial groups, blinding to hypotheses, and training in equipoise.^20^

Eligible participants were age 18 years or older and met criteria for MetS defined by the *Joint Interim Statement*.^1^ Exclusions were for medical safety (eg, cardiovascular risk, food allergies); logistical barriers (eg, inability to attend in-person meetings, no primary care physician); motivation (eg, unwilling or unable to change lifestyle); medical confounding (eg, diabetes, weight loss medications, oral corticosteroids); and mental health (eg, major depression, substance abuse, cognitive impairment).^17^

Potential participants, recruited in 4 waves of 150 participants per wave, were identified through electronic medical records (334 [54%]), self-referred through social or mass media advertisements (247 [40%]), or from other miscellaneous sources (37 [6%]). Screening required attendance at an information session that described the time needed for lifestyle change and provided time to consider personal pros and cons of participation (eMethods 1 in Supplement 2).^21^ The baseline examination included clinic visits for medical eligibility and a run-in for exposure to trial assessment demands. In all, 14 817 adults were screened during a 2.5 year period.

Eligible participants with confirmed MetS and continued interest were randomized by an unblinded statistician in a 1:1 ratio, stratified by site, and followed up for 24 months. COVID-19 restrictions required a pause in recruitment, remote assessments, and/or safety precautions for in-person examinations. Retention was maximized by obtaining alternative contacts, developing relationships with participants, minimizing transportation burden, reimbursing for assessment time, and conducting partial assessments with potential dropouts.

### Interventions

#### Core Elements in Both Study Groups

Both groups received education plus a Fitbit (Google Inc) activity monitor. The hypothesis was that education and activity monitoring were necessary but not sufficient to produce sustained MetS remission.

#### Comparator Group

Evidence-based education on MetS including nutrition, physical activity, and stress management^12,22,23^ was sent in 24 monthly tip sheets. A trained local coordinator made telephone contact every 3 months to provide trial updates and to answer queries. Participants were referred to tip sheets for lifestyle questions, and to their primary care practitioner for medical questions. Drift from the telephone contacts protocol was remediated in monthly supervisor meetings.

#### Intervention Group

A lifestyle program was developed over 15 years in which habit formation became the key strategy and components; length, mode of administration, and dose were also determined.^24^ The rationale for a mindfulness component was to enhance motivation during early habit formation by focusing on the immediate benefits of vegetables at meals and daily brisk walks.

Dietitians conducted individual 60-minute dietary consultations at baseline and 6 months. The 6-month intervention was delivered in 19 in-person group meetings of 90 minute duration with 15 participants per group. The meetings were co-led by a psychologist and a dietitian, and supported by a lay health coach who provided logistics assistance, including weighing participants. The rationale for group treatment was to encourage peer support and facilitate vicarious learning through watching peers make changes. Content progressed from eating (ie, nutrition, what to eat, how to cook, and emotional/opportunistic eating) to sustaining change with physical activity and ongoing contacts with health-conscious peers. The goal was to make 4 simple habits an automatic part of daily routine: (1) vegetables at meals^25^; (2) daily brisk walks^26,27^; (3) sensory awareness of smells, colors, and tastes^28,29,30^; and (4) emotion regulation by pausing before reacting to stress or opportunistic eating.^28,29,30^ Correlations between each simple habit and the complex risk factor they were intended to represent ranged from 0.18 to 0.44.^31^ The strategy for new habit formation was to repeat a new habit in response to repeated daily cues until the habit became automatic.^32,33^ This was accomplished in group meetings with an ecologically valid format that mirrored daily activities. Participants walked together, practiced sensory awareness, prepared a vegetable dish, and ate together while increasing sensitivity to immediate benefits of new habits.^24,34^ At home, daily repetition of all 4 habits was fostered by peer-to-peer accountability using the Fitbit Friends/Community apps. COVID-19 restrictions resulted in temporary shifts to remote meetings and implementation of safety protocols for in-person groups. Drift from the protocol was remediated in monthly supervisor meetings.

In the 18 months after the intervention, participants were offered monthly support meetings but no new content. These meetings combined all participants randomized to the intervention at a site, and were given in person, hybrid, or remotely.

#### Fidelity Monitoring

Intervention fidelity assessed delivery and receipt.^35^ In the comparator group, delivery was assessed by rate of successful tip sheet mailings and 3-month telephone calls; the extent to which materials were read was not tracked. Receipt was assessed by frequency of problems with tip sheets or the Fitbit reported during routine telephone calls. In the intervention group, delivery was assessed by observer checklists for delivery of required components at each group meeting, and by content and process variables rated from 20% of randomly selected video recordings from each site and recruitment wave. Receipt was assessed by group attendance and a comprehension questionnaire completed by participants after each group meeting.

### Outcomes

Self-reported sociodemographic information was collected using defined options that included an “other” category with a space to write-in a response. Race and ethnicity allowed for assessment of balance between groups, subgroup differences, and generalizability of results.

The primary outcome was remission of MetS at 24 months, defined as less than 3 cardiometabolic risk factors meeting MetS diagnostic criteria^1^: elevated blood pressure (systolic ≥130 mm Hg or diastolic ≥85 mm Hg or antihypertensive drug therapy); abdominal obesity (waist circumference ≥102 cm for male and ≥88 cm for female individuals); elevated triglycerides (≥150 mg/dL or drug treatment; to convert mg/dL to mmol/L, multiply by 0.0113); elevated fasting glucose (≥100 mg/dL or drug treatment; to convert mg/dL to mmol/L, multiply by 0.0555); and low HDL cholesterol (<40 mg/dL for male and <50 mg/dL for female individuals, or drug treatment; to convert mg/dL to mmol/L, multiply by 0.0259). Blood was drawn after a 12-hour fast and sent to an independent and blinded laboratory for assessment of HDL cholesterol, triglycerides, and glucose. Blood pressure was the average of 3 measurements after a 5-minute rest.^36^ Waist circumference was the average of 2 assessments on skin at the iliac crest.^37^ At baseline and 6 months, a report was sent to participants and, if requested, their primary care practitioner.

Secondary clinical outcomes included MetS components; glycated hemoglobin (HbA_1c_); MetS severity^38^; weight; and body mass index (BMI; calculated as weight in kilograms divided by height in meters squared). Targets of the intervention included self-reported vegetable intake (US National Cancer Institute Vegetable Screener^39^); moderate-intensity physical activity minutes per week and daily steps (accelerometer^40,41^); self-reported sensory awareness and emotion regulation (Observe and Non-React Facets of 5-Facet Mindfulness Questionnaire^42^); and self-reported automatic daily habits (Self-Report Habit Index^43^). Intervention satisfaction at 24 months was assessed as the percentage of respondents who would “definitely recommend this program to family and friends.”^17^ Outcome measures with scoring and cut points were previously published.^17^

Blinded telephone calls every 3 months ascertained vital status, adverse events, health care utilization, and nontrial treatments. A 15-month assessment of MetS was conducted for retention purposes only. Safety was evaluated monthly by a safety committee and monitored by an independent data and safety monitoring board. Data were collected and transferred to the central secure database using 4 electronic systems, ie, Snap Survey online data capture forms; CentrePoint for accelerometry data; Quanum Portal for laboratory results; and Fitabase for Fitbit data.

### Statistical Analysis

#### Sample Size

For the primary outcome (MetS in remission at 24 months), we used 6 assumptions: (1) MetS remission in intervention group, 40% or more; MetS remission in comparator group, 20% or less; (2) minimum power of 90%; (3) significance level of *P* < .05; (4) mixed logistic model with clustering in 1 group^44^; (5) intraclass correlation of 0.15 estimated from prior reviews^45,46^; and (6) 20% loss to follow-up. The 40% remission in the intervention was clinically significant based on comparability, with 50% adherence to drug therapy^47,48^ and a 35% or greater success rate for US Food and Drug Administration weight loss studies,^49^ and accounted for erosion from the 54% MetS remission observed in the proof of concept.^24^ The 20% MetS remission in the comparator was derived from the average 15% MetS remission in education controls in lifestyle interventions for MetS with follow-up periods of 1 year or more.^50,51,52,53,54,55^ These assumptions produced a sample size of 572, rounded upward to a sample of 600, and then increased to 618 to avoid turning away those in the process of enrolling when the target of 600 was reached.

#### Statistical Analysis Plan

The statistical analysis plan is available in the trial protocol in Supplement 1. The primary analysis compared groups on MetS remission at 24 months using mixed-effects logistic regression drawn from the logistic version of models by Candlish,^56^ included a random effect for partial clustering, and adjustment for prespecified baseline fixed-effect covariates age, sex, race, ethnicity, education, income, geographic site, BMI, and number of comorbidities.^44,57^ Secondary analyses compared groups on outcomes at 6 and 24 months in a similar manner. Analyses were performed using SAS, version 9.4 (SAS Institute) from March 2024 to May 2025.

The prespecified approach to missing data (trial protocol in Supplement 1) was to identify the pattern of missing data and link it to an appropriate imputation plan. Missing data were judged to be *missing not at random* because missingness at 24 months could not be predicted by any baseline or 6-month measures. The primary imputation model was last observation carried forward, justified by a failure to support the concern that it is an optimistic estimate of treatment benefit.^58^ Missingness could not be explained by status on treatment targets such as MetS, weight, or BMI assessed posttreatment at 6 months. Missing data were replaced with the most recent assessment from the 15-month, 6-month, or baseline examination. Sensitivity analyses using pattern mixture models for a series of tipping points^59^ are presented in eMethods 3 in Supplement 2.

## Results

**Figure 1.**
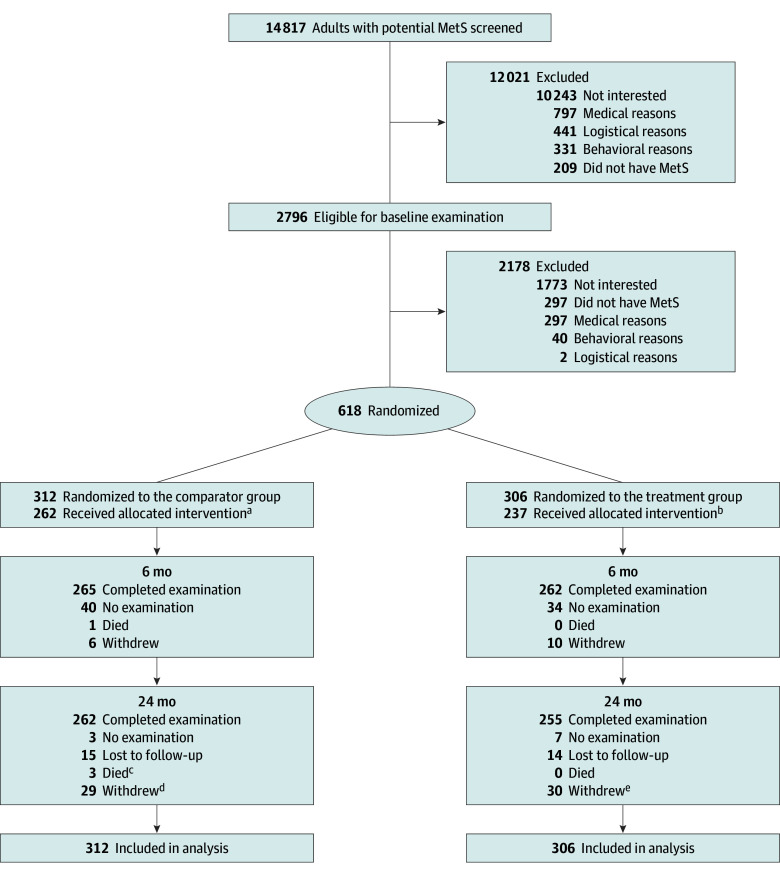
Consort Diagram of Study Participants in Study of Sustained Remission of Metabolic Syndrome (MetS) ^a^Received 100% of mailings in the first 6 months. ^b^Attended at least 50% of sessions in the first 6 months. ^c^Cause of death: stage IV lung cancer, stage IV liver cancer, COVID-19. ^d^Unknown, 19; relocation, 5; logistics, 4; and health, 1. ^e^Unknown, 11; health, 10; logistics, 7; relocation, 1; and administrative, 1.

The analysis included 618 participants (mean [SD] age, 55.5 [11.0] years; 468 female [74.7%] and 150 male [24.3%] individuals), who were randomized, 306 (49.5%) to the intervention and 312 (50.5%) to the comparator (Figure 1). The 24-month follow-up period was completed by 517 participants (83.7%). The loss, withdrawal, or death rate of 16.3% (101 of 618) at 24 months was similar across groups and lower than the 20% assumed in sample size calculations. Table 1 presents comparability between groups at baseline. Demographic, clinical, and lifestyle characteristics did not differ significantly between groups.

**Table 1.** Baseline Participant Characteristics, in Total Cohort and by Study Group

Characteristic	Total (N = 618)	Intervention group (n = 306)	Comparator group (n = 312)
**Demographic, No. (%)**			
Age, mean (SD), y	55.5 (11.0)	55.5 (11.0)	55.5 (11.0)
Female	468 (75.7)	239 (79.1)	229 (73.4)
Male	150 (24.3)	67 (21.9)	83 (26.6)
Race (self-reported)			
African American/Black	106 (17.1)	53 (17.3)	53 (17.0)
Asian	9 (1.5)	3 (1.0)	6 (1.9)
White	456 (73.8)	225 (73.5)	231 (74.0)
Other^a^	47 (7.6)	25 (8.2)	22 (7.1)
Hispanic/Latine ethnicity	60 (9.7)	33 (10.8)	27 (8.7)
Less than college degree	250 (40.5)	127 (41.5)	123 (39.4)
Married or cohabitating	381 (61.7)	191 (62.4)	190 (60.9)
Family income <$90 000	288 (45.6)	145 (47.4)	143 (45.8)
**Clinical, mean (SD)** ^b^			
Waist circumference, cm	115.1 (14.0)	114.7 (14.1)	115.5 (14.0)
Systolic blood pressure, mm Hg	128.1 (15.4)	127.6 (15.6)	128.6 (15.1)
Diastolic blood pressure, mm Hg	83.1 (10.0)	82.4 (10.1)	83.8 (9.9)
Triglycerides, mg/dL	175.3 (96.1)	179.6 (88.4)	171.1 (103.1)
Fasting glucose, mg/dL	100.0 (12.6)	100.0 (12.2)	100.1 (13.0)
HDL cholesterol, mg/dL	47.0 (11.1)	47.4 (11.8)	46.6 (10.5)
Medications, No. (%)			
Blood pressure	398 (64.4)	191 (62.4)	207 (66.4)
Triglycerides	24 (3.9)	15 (4.9)	9 (2.9)
Glucose (metformin)	59 (9.6)	31 (10.1)	28 (9.0)
HDL cholesterol	21 (3.4)	12 (3.9)	9 (2.9)
Body mass index^c^	36.5 (7.0)	36.3 (7.1)	36.7 (6.9)
Weight, kg	102.0 (21.8)	100.5 (21.6)	103.4 (22.0)
LDL cholesterol, mg/dL	116.5 (33.9)	116.5 (34.8)	116.5 (33.2)
Hemoglobin A_1c_,^d^ %	5.6 (0.4)	5.6 (0.4)	5.6 (0.4)
MetS severity^e^	0.97 (0.52)	0.98 (0.53)	0.96 (0.51)
Current smoking, No. (%)	30 (4.9)	15 (4.9)	15 (4.8)
**Quality of life** ^f^			
Perceived health	63.9 (17.4)	63.3 (17.0)	64.6 (17.8)
Energy/vitality	56.0 (18.4)	55.4 (19.1)	56.5 (17.7)
Depressive symptoms	2.3 (2.3)	2.4 (2.4)	2.3 (2.2)
Perceived stress	19.9 (6.7)	20.2 (6.8)	19.6 (6.6)
**Lifestyle targets of intervention** ^g^			
Vegetable intake, servings/d	3.0 (2.2)	3.1 (2.2)	2.9 (2.1)
Moderate-intensity physical activity, min/wk	104.9 (97.6)	112.7 (102.4)	97.2 (92.1)
Daily steps	4363 (2228.0)	4583.6 (2327.7)	4146.7 (2107.8)
Sensory awareness	3.5 (0.6)	3.5 (0.6)	3.5 (0.6)
Emotion regulation	3.5 (0.6)	3.5 (0.6)	3.5 (0.6)
Automatic habits			
Brisk walks	2.8 (1.0)	2.8 (0.9)	2.8 (1.0)
Vegetables at meals	2.8 (1.0)	2.8 (0.9)	2.8 (0.9)
Notice sensory experiences	3.7 (0.9)	3.7 (0.9)	3.8 (0.8)
Pause before reacting	3.3 (0.8)	3.3 (0.8)	3.3 (0.8)

Abbreviations: HDL, high-density lipoprotein; LDL, low-density lipoprotein; MetS, metabolic syndrome.

^a^ Included American Indian/Alaska Native, Native Hawaiian/other Pacific Islander, multiracial, other, and no response.

^b^ MetS cut points: waist circumference, ≥102 cm (male) and ≥88 cm (female); systolic blood pressure, ≥130 mm Hg or taking antihypertensive medication (for mm Hg to kPa, multiply by 0.133); diastolic blood pressure, ≥85 mm Hg or taking antihypertensive medication (for mm Hg to kPa, multiply by 0.133); triglycerides, ≥150 mg/dL or treatment for elevated triglycerides (for mg/dL to mmol/L multiply by 0.0113); glucose, ≥100-125 mg/dL or taking metformin (upper limit excludes diabetes; for mg/dL to mmol/L, multiply by 0.0555); HDL cholesterol, <40 mg/dL (male) and or <50 mg/dL (female), or taking medication for low HDL (for mg/dL to mmol/L, multiply by 0.0259).

^c^ Weight (kg) divided by height in meters squared (overweight, 25 to <30; obese, ≥30).

^d^ Normal, <5.7%; prediabetic, 5.7%-6.4%; diabetic, ≥6.5%.

^e^ Total cohort range, −0.51 to 2.60 (higher scores indicate more severe).

^f^ Assessment tools: perceived health, energy/vitality, Short Form-36, (scale, 0-100; higher scores indicate better quality); depressive symptoms, Patient Health Questionnaire-8 (scale 0-24; higher scores indicate more symptoms); and perceived stress, Perceived Stress Scale (scale, 0-56; higher scores indicate more stress).

^g^ Assessment tools: vegetable intake (servings/d), National Cancer Institute All Day Screener, vegetable subscale (higher scores indicate more servings); physical activity, Actigraph WGT3X-BT accelerometer (guidelines are 150 min/wk of moderate-intensity physical activity and 7500 steps/d); sensory awareness and emotion regulation, 5-Factor Mindfulness questionnaire; and automatic habits, Self-Report Habit Index (scale 1-5; higher scores indicate more automatic habit).

**Figure 2.**
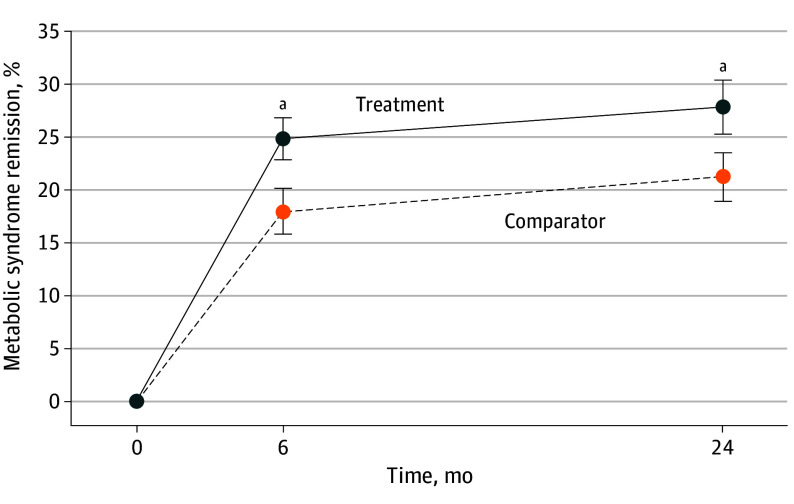
Metabolic Syndrome Remission, by Study Group and Time Remission at 6 months: adjusted odds ratio, 1.64 (95% CI, 1.07-2.53; *P* = .03); and remission at 24 months: adjusted odds ratio, 1.46 (95% CI, 1.01-2.14; *P* < .05). Adjustments for clustering and prespecified covariates age, sex, race, ethnicity, education, income, geographic site, body mass index, and number of comorbidities. ^a^*P* < .05.

Figure 2 and Table 2 present MetS remission by group and time. At 6 months, the proportion of MetS remissions was higher in the intervention (76 of 306 [24.8%]) than in the comparator (56 of 312 [17.9%]); adjusted odds ratio [OR] 1.64; 95% CI, 1.07-2.53; *P* = .03). At 24 months, the proportion of MetS remissions was higher in the intervention (85 of 306 [27.8%]) than in the comparator (66 of 312 [21.2%]) group, with an adjusted OR of 1.46 (95% CI, 1.01-2.14; *P* < .05). Approximately 15 patients need to receive the intervention to achieve 1 MetS remission. For completeness, eFigure 1 in Supplement 2 shows MetS remission with 15-month data added, but its purpose was for retention only, and it had a high rate of missing data.

**Table 2.** Secondary Outcomes, by Study Group and Time

Outcome	Follow-up time					
	6 mo			24 mo		
	Intervention group	Comparator group	*P* value^a^	Intervention group	Comparator group	*P* value^a^
Participants, No.	306	312	NA	306	312	NA
Participants with complete data, No. (%)	262 (85.6)	265 (84.9)	NA	255 (83.3)	262 (84.0)	NA
**Clinical, mean (SD)**						
Metabolic syndrome remission, No./Total No. (%)	76/306 (24.8)	56/312 (17.9)	.03	85/306 (27.8)	66/312 (21.2)	.05
Waist circumference, cm	110.2 (14.7)	113.6 (14.1)	<.001	111.7 (15.1)	113.1 (14.0)	.17
Systolic blood pressure, mm Hg	125.7 (15.7)	125.3 (15.0)	.40	125.7 (15.9)	125.2 (15.7)	.30
Diastolic blood pressure, mm Hg	80.4 (10.9)	81.1 (10.9)	.78	80.0 (11.3)	80.7 (10.3)	.95
Triglycerides, mg/dL	154.0 (75.8)	159.9 (92.1)	.008	163.1 (93.5)	161.8 (101.2)	.52
Fasting glucose, mg/dL	97.9 (11.1)	100.4 (13.7)	.01	97.7 (13.5)	100.7 (15.5)	.04
HDL cholesterol, mg/dL	49.0 (11.7)	47.5 (10.3)	.13	49.5 (12.4)	48.4 (11.0)	.40
Body mass index	34.9 (7.2)	36.4 (7.0)	<.001	35.4 (7.3)	36.0 (7.0)	.20
Weight, kg	96.6 (21.9)	102.3 (21.9)	<.001	97.7 (22.0)	101.1 (21.9)	.22
LDL cholesterol, mg/dL	113.2 (34.7)	114.8 (34.1)	.39	110.0 (36.3)	112.7 (35.9)	.25
Hemoglobin A_1c_, %	5.5 (0.4)	5.6 (0.4)	.02	5.6 (0.4)	5.7 (0.4)	.16
Metabolic syndrome severity	0.7 (0.6)	0.8 (0.6)	<.001	0.7 (0.7)	0.8 (0.6)	.06
Medications No./Total No. (%)						
Blood pressure	186/306 (60.8)	209/312 (67.0)	.07	192/306 (62.7)	223/312 (71.5)	.02
Triglycerides	13/306 (4.3)	9/312 (2.9)	.39	13/306 (4.3)	9/312 (2.9)	.39
Glucose (metformin)	24/306 (7.8)	26/312 (8.3)	.88	29/306 (9.5)	35/312 (11.2)	.51
HDL cholesterol	10/306 (3.3)	9/312 (2.9)	.82	10/306 (3.3)	9/312 (2.9)	.82
**Quality of life, mean (SD)**						
Perceived health	70.6 (16.9)	67.2 (17.7)	<.001	68.4 (17.2)	67.3 (17.8)	.13
Energy/vitality	61.5 (19.3)	56.3 (18.6)	<.001	59.6 (19.9)	57.8 (19.5)	.12
Depressive symptoms	2.4 (3.0)	2.8 (3.1)	.07	2.9 (3.4)	2.7 (3.0)	.71
Perceived stress	19.1 (6.7)	20.0 (7.1)	.006	19.4 (7.2)	19.4 (7.2)	.36
**Lifestyle targets of intervention, mean (SD)**						
Vegetable intake, servings/d	4.8 (3.5)	3.5 (2.6)	<.001	4.0 (4.0)	3.4 (2.4)	.03
Moderate-intensity physical activity, min/wk	127.5 (122.6)	100.4 (103.1)	.02	122.5 (164.3)	101.3 (123.6)	.18
Daily steps	5356.0 (2956.4)	4316.2 (2417.0)	< .001	4822.6 (2784.6)	4157.8 (2393.2)	.02
Sensory awareness	3.8 (0.6)	3.6 (0.7)	<.001	3.8 (0.7)	3.6 (0.6)	< .001
Emotion regulation	3.5 (0.6)	3.5 (0.6)	.87	3.6 (0.6)	3.5 (0.6)	.48
Automatic habits						
Brisk walks	3.3 (0.9)	3.0 (0.9)	<.001	3.3 (0.9)	3.0 (1.0)	.001
Vegetables at meals	3.4 (0.9)	3.2 (0.9)	.004	3.4 (1.0)	3.3 (1.0)	.12
Notice sensory experiences	3.8 (0.8)	3.8 (0.8)	.30	3.9 (0.8)	3.8 (0.8)	.20
Pause before reacting	3.4 (0.8)	3.3 (0.8)	.12	3.4 (0.8)	3.3 (0.9)	.94
Intervention satisfaction, No./Total No. (%)	NA	NA	NA	172/218 (78.9)	97/205 (47.3)	<.001

Abbreviations: HDL, high-density lipoprotein; LDL, low-density lipoprotein; NA, not applicable.

^a^ Adjusted for clustering and the prespecified baseline covariates of age, sex, race and ethnicity, education, income, geographic site, body mass index, and number of comorbidities.

Secondary end points are presented in Table 2 as group-specific means or percentages with adjusted *P* values for differences, and also in the eMethods 5 in Supplement 2 as adjusted effect size estimates of the difference between groups (aES), with 95% CIs. Among the clinical end points at 6 months, the intervention was superior to the comparator on waist circumference, triglycerides, fasting glucose, BMI, weight, HbA_1c_, MetS severity, and quality of life. At 24 months, statistically significant differences between the intervention and comparator groups were sustained only for fasting glucose (97.7 mg/dL vs 100.7 mg/dL; aES, −2.74; 95% CI, −5.31 to −0.16; *P* = .04). The intervention was associated with a reduction in blood pressure medication over the comparator (192 of 306 [62.7%] vs 223 of 312 [75.5%], respectively; aES, −0.06; 95% CI, −0.103 to −0.009; *P* = .02).

**Figure 3.**
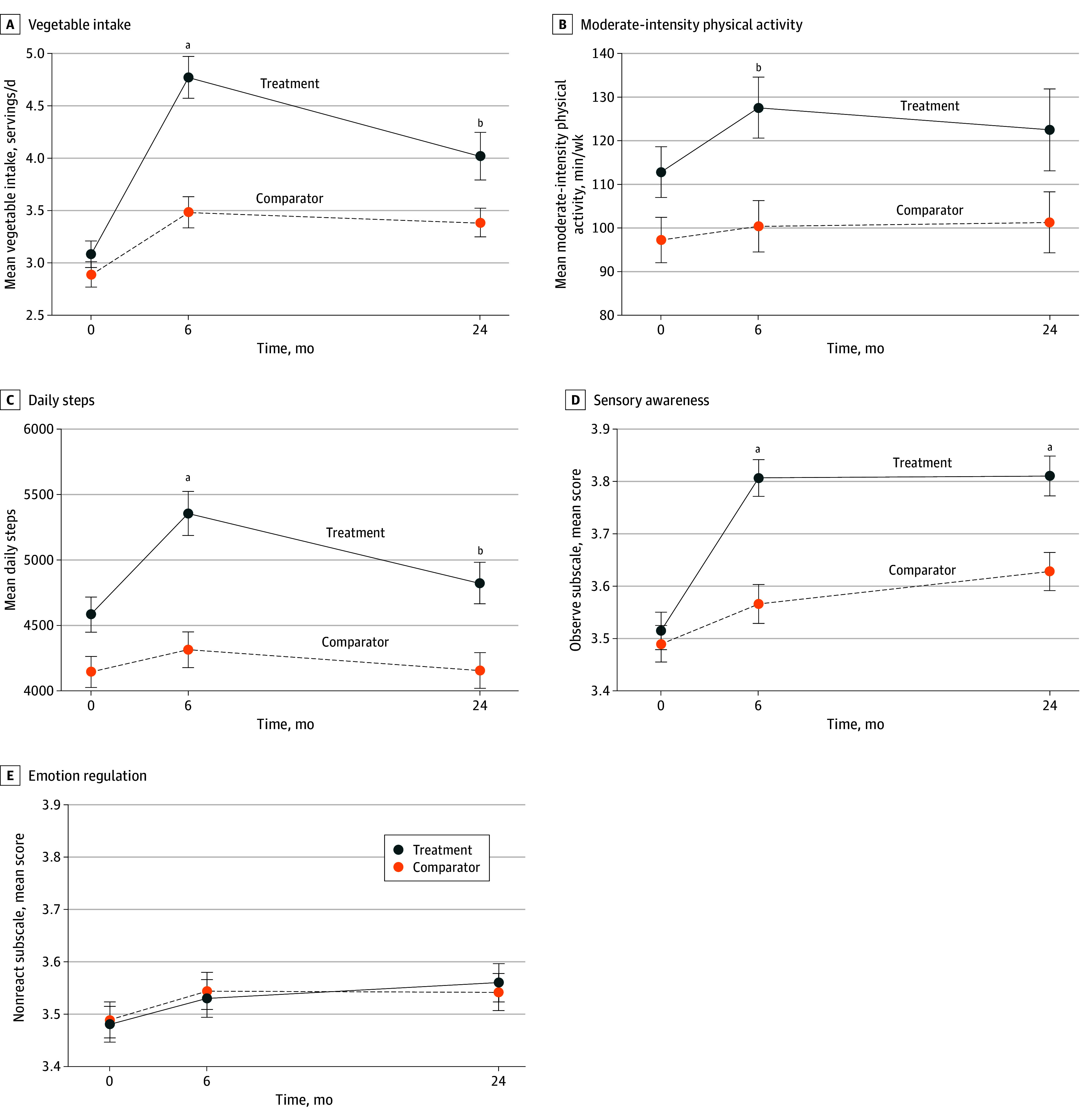
Lifestyle Intervention Targets, by Study Group and Time Statistical tests adjusted for clustering and prespecified covariates: age, sex, race, ethnicity, education, income, geographic site, body mass index, and number of comorbidities. Sensory awareness and emotional regulation assessed by the 5-Factor Mindfulness questionnaire as the average item response to a 5-point Likert scale, with higher scores representing more mindfulness. Range in total cohort: sensory awareness, 1.6-5.0, and emotion regulation, 1.4-5.0. ^a^*P* < .001. ^b^*P* < .05.

Table 2 and Figure 3 present lifestyle intervention targets, by group and time. At 6 months, the intervention group was significantly different from the comparator on all lifestyle factors except emotion regulation. At 24 months, significant differences were sustained for vegetable intake (4.0 vs 3.4; aES, 0.52; 95% CI, 0.04-0.99; *P* = .04), daily steps (4822.6 vs 4157.8; aES, 432.5; 95% CI, 66.4-798.6; *P* = .02), and sensory awareness (3.8 vs 3.6; aES, 0.17; 95% CI, 0.08-0.26; *P* < .001). Among daily habits, brisk walks and vegetables at meals were significant at 6 months and sustained at 24 months for brisk walks only. Intervention satisfaction (ie, percentage who would definitely recommend this intervention) was higher in intervention than in the comparator group (172 of 218 [78.9%] vs 97 of 205 [47.3%]; *P* < .001).

Subgroup analyses evaluated odds of MetS remission for intervention vs comparator groups within prespecified subgroups at 6 and 24 months (eFigure 2 in Supplement 2). There was no observed heterogeneity of treatment effect across subgroups.

Intervention fidelity was excellent. In the comparator group, 84% of the participants received 100% of the mailings in the first 6 months, 92% of the 3-month telephone calls were completed, and 292 participants (94%) reported no problems. In the intervention group, 98% (299 of 306 participants) and 70% (182 of 262) completed individual dietary consultations at baseline and 6 months, respectively. During the 6-month intensive phase, delivery of required content was accomplished in 94% of meetings with an average process score of 28.6 of 31.0 points. Each participant attended an average of 67% of group meetings and comprehension of content averaged 4.4 on 5-point scale. Average attendance at the follow-up support meetings between 6 and 24 months was 37%.

No safety concerns were reported. There were 437 blinded adjudications that were classified as 115 serious adverse events, 290 adverse events, and 32 nonevents. No events were determined to be related to trial participation.

## Discussion

MetS is growing and is fundamentally affected by lifestyle. Lifestyle interventions have achieved MetS remission but sustaining it after treatment ends is a challenge.^60^ The evolving science of sustained behavior change suggests that unique strategies are needed to achieve sustainability,^61^ one of which is new habit formation.^32,33^ ELM was designed to determine whether a habit-formation program could augment education and activity monitoring to achieve sustained MetS remission over a long-term follow-up period after treatment.

The primary result was that 28% of participants in the intervention vs 21% in the comparator group were in MetS remission at 24 months. The intervention produced 25% MetS remission after its intensive 6-month phase and sustained it without treatment for the next 18 months. This result is meaningful when compared to the average 22% MetS remission observed in lifestyle trials of similar participants who received some type of ongoing treatment over the course of their follow-up.^14,15,16^ Sustained MetS remission was supported by sustained improvement in fasting glucose. Although lifestyle interventions for MetS have been shown to improve glucose,^60^ ELM is the first to show sustained improvement after active treatment ended. Differences between groups in weight loss and its correlates at 6 months were no longer significant at 24 months, which suggests that they were more important for initiation than sustaining MetS remission—an observation we made previously.^24^

The most convincing support for lifestyle change was in daily steps. At 24 months, participants in the intervention group walked an average of 4823 daily steps, that is, 665 steps more than those in the comparator. Although less than the goal of 7500 steps (at which health benefits plateau^62^) and less than the definition of sedentary behavior (≤5000 steps daily),^63^ recent meta-analyses found that walking more than 4000 steps daily reduced risk of all-cause, cardiovascular, and cancer mortality,^26,64^ and daily steps were better predictors of mortality than intensity of physical activity.^26^ Significant differences between groups were observed for sensory awareness and the habit of daily brisk walks. Taken together, the simple habit of daily brisk walks for their immediate benefits may be a promising alternative for sustainability than setting more intensive goals to achieve future health gains.

Participants in the comparator group underwent greater MetS remission than expected. Receiving only a Fitbit activity monitor and 24 monthly mailed education tip sheets, 21% achieved MetS remission at 24 months, higher than the average 15% remission observed in previous education controls.^50,51,52,53,54,55^ This could be explained by the high level of motivation in the ELM cohort. Screening required potential participants to be informed about the time needed to make lifestyle changes, to have sufficient time to reflect on their personal pros and cons, and to complete a run-in exposing them to assessment demands. Those who remained interested may have had sufficient motivation to make lifestyle changes with minimal intervention.^65^ Activity monitoring, by itself, has been shown to increase daily steps.^66^

Participants in the intervention underwent less MetS remission than hypothesized. It is possible that COVID-19 restrictions during the trial, which required paused, hybrid, and socially distanced meetings, may have diluted its effects. However, 28% MetS remission has clinical relevance when compared to the 1711 participants with MetS in the Diabetes Prevention Program (DPP).^16^ Both trials had strict eligibility to protect internal validity, with DPP enrolling only 2% of screened^67^ and ELM enrolling 4% of screened patients. The trials had almost comparable participants with MetS and featured intensive 6-month lifestyle interventions with 18-month follow-ups. However, DPP featured intensive follow-up after initial treatment, including ongoing group courses, motivational campaigns, and as many individual contacts as needed,^68^ whereas ELM offered only voluntary support contacts to large groups of participants. MetS remission at 24 months was 11% in the DPP lifestyle group compared to 28% in the ELM intervention group. Thus, ELM achieved greater long-term remission than DPP despite having stopped the intervention.

### Strengths and Limitations

Among this trial’s strengths are that ELM fills the gap left by too few efficacy trials and inadequate follow-up periods as identified in systematic reviews of lifestyle interventions for MetS.^60,65,69,70^ ELM featured rigorous methods for single-blind behavioral efficacy trials,^20^ including progressive development of treatment,^24^ expansion of blinding to minimize single-blind bias,^20^ and priority on high retention over a long follow-up. The cohort was large and diverse, recruited from 5 geographic areas in the US and with representation by sex, race, ethnicity, education, and rural residence. The primary result of 28% MetS remission observed 18 months after the end of treatment compares favorably to past trials of similar patients receiving ongoing intervention, is supported by improvements in secondary outcomes, did not differ significantly in any subgroups, and incurred no safety risks.

Limitations must be considered.^71^ The result was statistically significant but modest. The 24-month OR of 1.46 was surrounded by a wide 95% CI of 1.01 to 2.14 in a comparison of 2 active treatments, only one of which ended after 6 months. Nonspecific attention may have explained results for treatments that differed in intensity of contact. The trial may have been overpowered to detect small differences because assumptions about losses and the intraclass correlation in sample size calculations were more conservative than observed. The effect size may have been diluted by the differential impact of COVID-19 restrictions on the in-person meetings in the intervention group, which did not affect the remote comparator. An intensive in-person program may be difficult to implement and not cost-effective, suggesting the value of exploring fully remote versions.

## Conclusions

This randomized clinical trial found that sustained MetS remission is possible using the ELM strategy of simplifying targets and motivating integration of improved lifestyle habits into the daily routine by focusing on immediate benefits. Interventions that produce sustained health benefits over time are needed^60^; however, targeting lifestyle alone may limit the potential strength of an intervention for MetS. The new glucagon-like peptide-1 obesity drugs reduce caloric intake—only during treatment^72^—and appear to act in synergy with lifestyle interventions.^73,74^ It is compelling to consider the success and cost-effectiveness that could result from combining a drug that works while on treatment with a lifestyle intervention that works after treatment is discontinued. At a more basic level, changes in the socioecological systems associated with MetS are needed.^75^ However, until these basic changes are made, the prevalence of MetS continues to rise and evidence-based interventions are needed urgently.

## Notes

Supplement 1. Trial Protocol

Supplement 2. eMethods 1. Informed Consent: Slides for Information Session eMethods 2. Consent Form eMethods 3. Sensitivity Analyses eMethods 4. Data Sharing and Dissemination eMethods 5. Table 2 (Addition) Secondary Endpoint eFigure 1. Metabolic Syndrome Remission By Trial Arm and Time, including the 15-month assessment eFigure 2. Prespecified Baseline Subgroups: Odds of Metabolic Syndrome Remission At 6 and 24 Months

Supplement 3. Nonauthor Collaborators

Supplement 4. Data Sharing Statement
