# Supplementary material for: Lifestyle Intervention for Sustained Remission of Metabolic Syndrome: A Randomized Clinical Trial
Source: JAMA Intern Med. 2025 Nov 9;186(1):67–77. doi: 10.1001/jamainternmed.2025.5900 (PMC12598583; doi:10.1001/jamainternmed.2025.5900)
Supplement: Supplement 1. — Trial Protocol [file jamainternmed-e255900-s001.pdf]

Trial Protocol

The **E**nhanced **L**ifestyles for **M**etabolic Syndrome (**ELM**)

Trial Protocol

April 12, 2023

(Version 12)

TABLE OF CONTENTS

	Page
HISTORY	3-4
BACKGROUND	5
SPECIFIC AIMS	6-7
TRIAL DESIGN	7-8
EQUIPOISE AND BLINDING	8
ELIGIBILITY	9-10
RECRUITMENT AND RANDOMIZATION	11-12
INTERVENTIONS	12-15
OUTCOMES ASSESSMENT	15-18
RETENTION	18
DATA FLOW	18-20
SAMPLE SIZE	20-21
ANALYSIS PLAN	21-22
TRIAL ORGANIZATION	22
SAFETY MONITORING	22-25
REFERENCES	26-27

Trial Protocol History

DATE	DESCRIPTION	REVISED SECTION(S)
6/2/20	Original protocol	
7/21/20	Adverse events and unanticipated problems will be reported to the DSMB quarterly instead of monthly. This revision was recommended by the DSMB at the Annual DSMB meeting held on 6/30/20.	Safety Monitoring: Adverse Events<br>Figure 7
	The timing of in-person data collection visits are now aligned with the date of randomization. This differs from the previous alignment to dates associated with intervention delivery. The DSMB recommended this change at the Annual DSMB meeting held on 6/30/20	Outcomes Assessments: In-Person Follow-Up Visits
10/13/20	The Outcomes & Publications Committee extended the in-person follow-up study visit window from 6 weeks to 9 weeks. This was reported at the Steering Committee meeting on 10/13/20.	Outcomes Assessments: In-Person Follow-Up Visits
1/19/21	The Steering Committee approved the following exclusion criterion to be included in the ELM Trial protocol on 1/12/21:<br>• Participation in any clinical trial, until at least six months following the end of the intervention phase. Individuals in long-term follow-up (over 6 months post intervention) may be enrolled in ELM. Individuals in studies that are not clinical trials, such as observational studies or surveys may be enrolled in ELM.	Eligibility
1/26/21	The timing of in-person data collection visits is now aligned with the date of the last intensive phase session in the Group-Based arm. This differs from the previous alignment to dates of randomization. The DSMB endorsed this change at the DSMB meeting held on 1/25/20.	Outcomes Assessments
2/8/21	The original trial timeline was replaced with an updated timeline starting in Year 3.	Trial Design

4/20/21	The Executive Committee approved the exclusion criterion below at the 4/20/21 EC meeting.<br><br>Concurrent or planned participation in any other lifestyle intervention or behavioral weight loss program.	Eligibility
8/24/21	The window for baseline visits has been expanded to 3 months to allow sites to test for eligibility criteria (e.g. medications, labs, etc.) at the first visit. Sites can start baseline exams as soon as a potential participant has been identified.<br><br>The following sentence was added to the protocol, “For Wave 4, components of the metabolic syndrome may be collected up to 3 months before randomization.”	Screening
2/23/23	A updated trial timeline has been added in **Figure 2.**	Trial Design
2/24/23	15 month assessment data can be collected until the 21 month mark. 24 month assessment data can be collected from any wave until the day prior to trial closeout.	Outcomes Assessment
3/1/223	A updated Figure 3. The hypothesized pathway for the Group-Based program was added.	Interventions
4/12/23	Details added to the Maintenance phase (months 7-24)	The Group-Based Arm

BACKGROUND

The metabolic syndrome (MetS) is a preclinical warning sign for heart failure, diabetes, and cardiovascular disease. Together, these chronic conditions account for the majority of health care costs. The MetS is ***diagnosed by*** *the co-occurrence of at least 3 out of 5 cardio-metabolic risk factors: abdominal obesity, hypertension, hyperglycemia, hypertriglyceridemia, and low high-density lipoprotein cholesterol [1]. It quintuples the risk of diabetes, doubles the risk of cardiovascular disease [2], and is considered a state of prediabetes [3] and Stage A of heart failure [4].*

The MetS has increased in prevalence from one-quarter to one-third of Americans over the past 20 years [5]. Its fundamental cause is a lifestyle characterized by an imbalance of energy intake and output, exacerbated by chronic stress. The current health care system treats it with drugs, which are not taken in 50% of the patients and do not slow progression in the 50% that do take them. ***If as few as*** 40% of the 86 million Americans with the MetS made a sustained change in their lifestyle, it would result in a 60% reduction in health care costs and a savings of approximately $85 billion annually [6].

Prior lifestyle interventions that examined sustained, 2-year remission of the MetS have produced success in 10%-48% of participating patients. The greatest successes occurred in small experimental studies in *Mediterranean countries following high-intensity dietary interventions that cannot be easily implemented in clinical practice. In the United States, the Diabetes Prevention Program found a 2-year remission of 11.1% in the 549 participants randomized to the lifestyle arm who had the MetS at baseline [7]. All of the existing lifestyle interventions for the MetS targeted diet or diet plus physical activity. Chronic stress promotes the MetS directly* by triggering the release of glucocorticoids [8] and indirectly by *interfering with efforts to adopt healthy behaviors [9].* A 3-component lifestyle intervention targeting diet, physical activity, and chronic stress has never been evaluated for its ability to promote a sustained remission of the MetS.

With support from the William G. McGowan Charitable Fund, a multidisciplinary team of investigators from the Rush Department of Preventive Medicine developed the Eat Love Move (ELM) Lifestyle Program, a group-based lifestyle intervention for the MetS designed for implementation in the current health care system. Preliminary data suggested that it is plausible that the ELM program can achieve sustained 2-year remission of the MetS in over 50% of patients [10], can be implemented in a clinical setting, and can be delivered with high fidelity at a different national site.

The next step in this progressive program of research is to conduct a multi-site efficacy trial to test whether, and under what conditions, the ELM program can produce a sustained remission of the MetS when delivered across diverse national sites, and whether or not it offers a cost-effective alternative to the best option for lifestyle change that exists in current medical practice and is augmented by widely available wearable technology.

This multi-site trial is called the Enhanced Lifestyles for the Metabolic Syndrome (ELM) trial. Once the ELM trial began, the ELM Lifestyle Program was renamed the Group-Based Program. It will be compared to enhanced usual care, named the Self-Directed Program. The neutral names for the trial arms preserve equipoise and minimize reactivity to any pre-existing preferences.

SPECIFIC AIMS

The ELM trial aims to compare a Group-Based program to an enhanced usual care comparator called the Self-Directed program when implemented at the national level. A total of 600 participants are recruited from 5 geographic regions (Rochester NY, Wilkes-Barre PA, Chicago IL, Kansas City MO, and Denver CO) and randomized into 1 of the 2 arms in a ratio of 1:1. The primary evaluation will occur after 2 years and will focus on sustained remission of the metabolic syndrome (MetS), sustained change in health behaviors, medical status, quality of life, and the cost-effectiveness of each arm. These aims are operationalized as the following Specific Aims.

PRIMARY AIM 1

Compare the Group-Based and Self-Directed arms on remission of the metabolic syndrome at the 2-year follow-up. The hypothesis is that 2-year remission of the MetS will occur in ≥ 40% in the Group-Based arm and in ≤ 20% in the Self-Directed arm.

SECONDARY AIMS

AIM 2.

Compare the Group-Based and Self-Directed arms on the behavioral risk factor targets of the treatment, and other secondary outcomes, at 6, 15, and 24 months.

Aim 2a: Compare the Group-Based and Self-Directed arms on the proportion of participants achieving clinically significant improvement in behavioral risk factor targets of treatment: (1) 2 cups of vegetables/day; (2) at least 150 minutes of moderate intensity physical activity/week; (3) either the maximum score, or an increase of ≥ 1 standard deviation of baseline distribution, on the 3-facet Mindfulness Questionnaire; and (4) a score of ≥ 4 on each of the four domains of the Self-Reported Habit Index (diet, physical activity, emotion control, and sensory awareness).

Aim 2b: Compare the Group-Based and Self-Directed arms on the secondary outcomes of: (1) ≥ 5% loss of baseline weight; (2) kg of weight lost; (3) frequency of sugary beverage intake; (4) energy/vitality; (5) depressive symptoms; (6) social support for diet and exercise; (7) frequency of contacts with the health network; (8) perceived stress; (9) eating competence; (10) MetS components; (11) MetS severity; (12) Hemoglobin A1c; and (13) body mass index.

AIM 3.

Conduct a comprehensive health economic evaluation of the Group-Based and Self-Directed programs.

Aim 3a: Calculate the cost-effectiveness of the Group-Based relative to the Self-Directed programs. Cost-effectiveness is quantified using the Incremental Cost-Effectiveness Ratio and will reflect the cost of the programs in the two arms relative to their efficacy at promoting a sustained remission of the MetS.

Aim 3b: Model the projected clinical benefits and health care savings associated with dissemination of the Group-Based and Self-Directed programs.

Aim 3c: Compare the Group-Based and the Self-Directed programs on the number and dose of medications, and health care utilization (primary care visits, specialty care visits, emergency room visits, hospital admissions) at 2 years.

EXPLORATORY AIMS

AIM 4.

Evaluate the consistency of impact and delivery across national sites.

Aim 4a: Explore the efficacy of the Group-Based and Self-Directed programs in subgroups defined by: geographic site, type of recruitment, age, gender, race/ethnicity, education, income, and number of MetS components.

Aim 4b: Explore the fidelity of delivery of the Group-Based and Self-Directed programs by geographic site.

TRIAL DESIGN

This is a 6-year, 5-site, randomized behavioral efficacy trial of 600 participants with the MetS. Trial coordination is conducted by a research coordinating unit (RCU) and an intervention coordinating unit (ICU), located within the Department of Preventive Medicine at Rush University Medical Center in Chicago. Patients with MetS who are motivated to manage it by changing their lifestyle, and do not have any medical contraindications or logistical barriers, are recruited serially from each of the 5 participating clinical centers over 4 waves of 6-month recruitment periods. Each site is expected to recruit 30 participants/wave, and 120 participants over all 4 waves, to achieve a total of 600 participants in the trial. Recruitment proceeds in 3 steps to confirm medical, behavioral, and logistical eligibility. Once a site has recruited 30 participants, they are randomized in a ratio of 1:1 to the Group-based or Self-Directed arm. Randomization is stratified by site in permuted blocks. Outcome evaluation is conducted at baseline, at the end of the intensive phase of the intervention at approximately 6 months, halfway through the maintenance period at approximately 15 months, and at the end of follow-up at approximately 24 months. **Figure 1** presents the trial design. **Figure 2** presents the timeline for the 6-year trial including the set-up, serial recruitment in 4 waves, intervention, and maintenance. This revised trial timeline reflect s a 4-5 month suspension of in-person visits due to the Covid-19 pandemic.

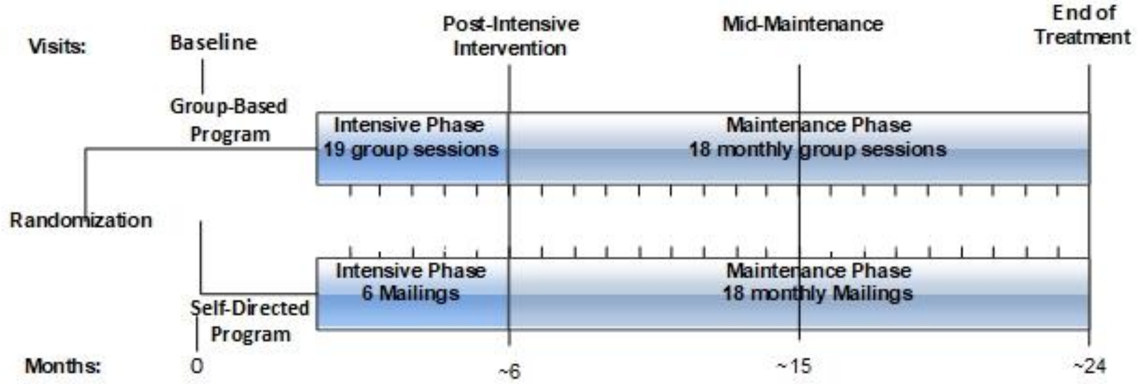

Figure 1. Trial design

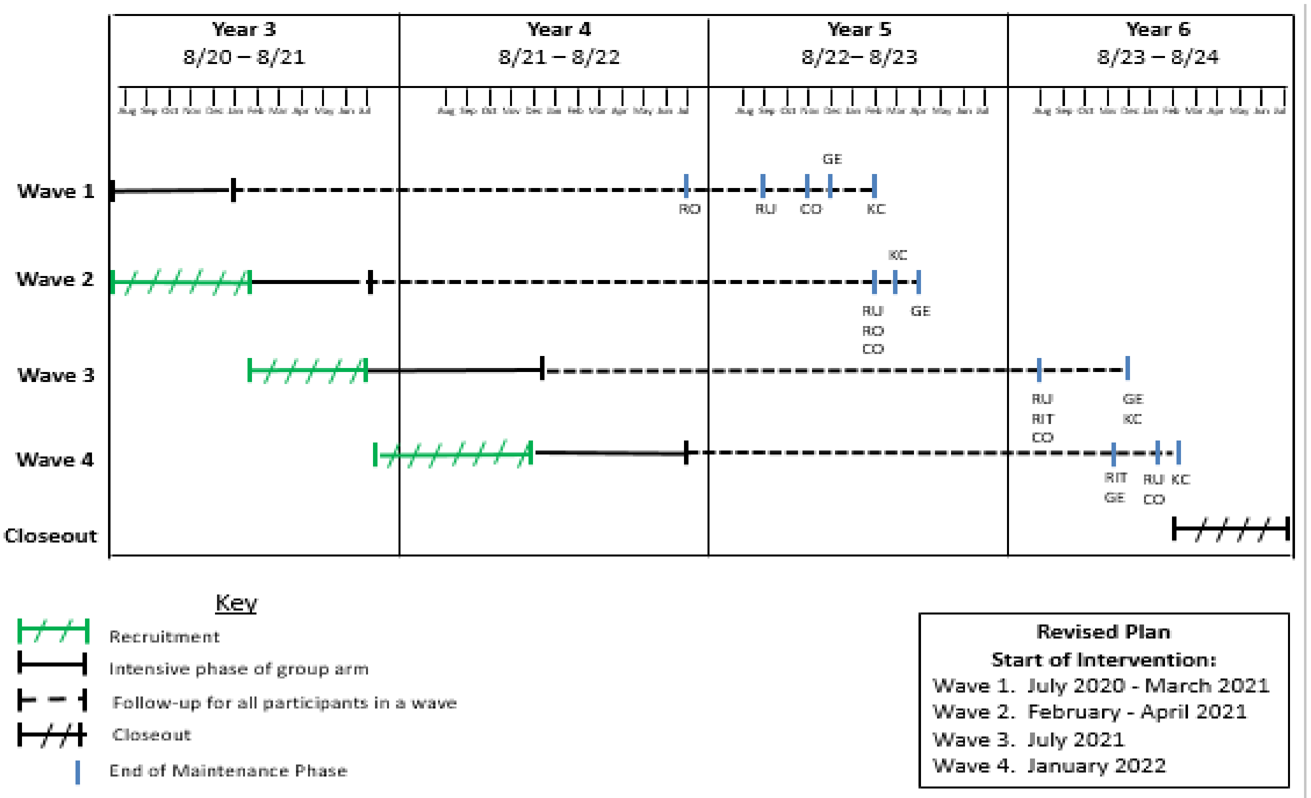

Figure 2. Revised trial timeline

EQUIPOISE AND BLINDING

Investigator preference for, and confidence in, a specific trial result can be an independent source of post-randomization bias triggering unintended interventions, treatment crossovers, co-intervention bias, and ascertainment bias. The potential for this bias in the ELM trial is minimized by targeting the mindset of the investigators. Their mindset has been shaped by presentations and discussions about objectivity as a hallmark of a scientific mindset, the need for discipline to keep preferences private, and the fallacy of expecting, in advance of a trial result, that one arm will be superior to the other. Trial arms have been renamed with neutral names: Group-Based (the treated group, previously the Eat Love Move (ELM) program); and Self-Directed (the enhanced care comparator). The blind is extended in trial operations to include: blinding to trial hypotheses but not to trial aims among all except the Principal Investigators; blinding to individual level treatment assignment among all but the interventionists; blinding of all but the senior biostatistician to the primary outcome of MetS; and blinding of interventionists to aggregated outcome data completion. Detection of risk of bias resulting from an absence of equipoise will be evaluated on an ongoing basis by assessing crossovers and co-interventions every 3 months and assessing differential attrition monthly.

ELIGIBILITY

To be eligible for participation, 600 individuals must meet the inclusion criteria and not meet any of the exclusion criteria.

Inclusion Criteria

1. Men and women with MetS, defined by the Joint Interim Statement of the IDF, NHLBI, AHA,WHF, IAS, IASO [1]. To meet these MetS criteria the participant has ≥ 3 of the following 5 components:
    a. Waist circumference of ≥ 102 cm for men and ≥ 88 cm for women;
    b. Triglycerides ≥150 mg/dL or treatment for elevated triglycerides;
    c. HDL cholesterol <40 mg/dL in men and <50 mg/dL in women, or treatment for low HDL;
    d. Systolic blood pressure ≥ 130 mmHg; diastolic blood pressure ≥ 85 mm Hg; or treatment for hypertension;
    e. Fasting plasma glucose 100-125 mg/dL (range inclusive) or on metformin.

2. Age 18 years or older.

Exclusion Criteria

Safety:

3. Unable to walk 2 consecutive blocks without assistance; pain, tightness, or pressure in the chest during physical activity; dizziness or lightheadedness without clearance from their physician.
4. History of an eating disorder in the past 5 years.
5. History of major cardiovascular illness: stroke; myocardial infarction; congestive heart failure > NYHA Class I; uncontrolled hypertension (SBP>180 or DBP > 110); unstable angina or an active prescription for sublingual nitroglycerin; or other cardiovascular illness deemed to limit safe participation.
6. Individuals with moderate hypertension (SBP ≥160 and ≤180; DBP ≥100 and ≤110) without written consent from their physician.
7. Severe food allergies or intolerances/preferences.

Logistics:

8. Unable to consistently attend group classes at a scheduled time due to lack of reliable transportation, schedule conflicts, travel, plans to relocate, upcoming surgery, etc.
9. No reliable access to the internet via a computer or mobile device.
10. Participant does not have a primary care physician and does not want one at the time of enrollment.

Motivation:

11. Unwilling, unable, or not ready to make lifestyle changes.
12. Unable or unwilling to complete the run-in protocol.

Risk of Attrition:

13. Unwilling to consent to randomization.

Special Population Needs:

14. Not fluent in English.
15. Visual or hearing impairment.

Confounding with Medical Treatment:

16. Current diagnosis of type 1 or type 2 diabetes, on any diabetes medications except metformin, or a screening A1c ≥7.0. Individuals with fasting blood glucose ≥ 126 or Hemoglobin A1c ≥6.5 and < 7.0 without confirmation from their physician that they do not have diabetes.
17. Inpatient treatment for a psychiatric condition within the past 6 months, or currently receiving treatment for schizophrenia or other serious psychiatric illness.
18. ≥ 30 days of oral corticosteroid use within the last year, history of solid organ transplant, or history of stem cell transplant.
19. Use of weight loss medications in the last 3 months, or unwilling to abstain from weight loss medications or supplements during the trial.
20. Pregnant women, planning a pregnancy in the next 24 months, given birth in the last 6 months, or currently breastfeeding.
21. History of bowel resection or bariatric surgery.
22. Any medical condition known to influence the etiology of MetS (e.g., uncontrolled hypothyroidism, endocrine hypertension, etc.).
23. Currently taking antiretroviral therapy.
24. Receiving dialysis
25. Cancer treatment within the last 6 months, excluding chemoprophylaxis or treatment for non-melanoma skin cancer
26. Participation in any clinical trial, until at least six months following the end of the intervention phase. Individuals in long-term follow-up (over 6 months post intervention) may be enrolled in ELM. Individuals in studies that are not clinical trials, such as observational studies or surveys, may be enrolled in ELM.
27. Concurrent or planned participation in any other lifestyle intervention or behavioral weight loss program.

Not Suitable for Group Treatment

28. Probable major depression, defined as a PHQ-8 score ≥ 10.
29. Problematic use of alcohol and/or recreational drugs, defined as ASSIST screening score of ≥27.
30. Cognitive impairment, defined by a Montreal Cognitive Assessment (MoCA) score of ≤25.

Carry-Over Effects

31. Previous participation in an ELM program or currently living with ELM participant.

RECRUITMENT AND RANDOMIZATION

Identification of Potential Participants

There are 3 key strategies used to identify potential participants.

1. Electronic Medical Records. The RCU has developed an algorithm for interrogating medical records. This algorithm identifies patients with probable MetS, without a diagnosis of diabetes, ≥18 years of age, and English speaking. All patients identified are sent an introductory letter with the option of opting out of receiving a subsequent recruiting phone call within 2 weeks of receipt of the letter.

2. Referral by Medical Provider. Referrals are accomplished in medical clinics by embedding research assistants within the clinic, posting recruitment materials in the exam rooms, and/or clinic-specific activities developed by a provider who serves as a local champion.

3. Self-Referral. Internet-based recruitment, advertisements, Facebook, and mass media visibility make it possible for interested potential participants to contact trial staff directly.

Sites have the flexibility to tailor their recruitment approaches to those that re likely to have the most success within their community.

Screening

Screening takes place in 3 steps.

Step 1: Initial Telephone Screening

Telephone screening provides a first pass at determining medical, behavioral, and logistical eligibility. Prospective participants are first asked about their interest in the trial. Then they self-report their status on MetS components and respond to a series of questions about medical, logistical, and safety eligibility. They are asked 2 specific questions about behavioral eligibility based upon willingness and ability to make changes in their lifestyle. Conditional upon eligibility after the screener is complete, prospective participants are asked about their continued interest in the trial and their willingness to attend an Information Session to get more information.

Step 2: Information Session

The Information Session aims to provide potential participants with a better understanding of what lifestyle change entails and what is required for participation in a randomized clinical trial. The approach is not a "hard sell" to enhance participation, but rather an effort to obtain true informed consent by giving the prospective participant the opportunity to engage in a thoughtful consideration of the pros and cons of participation, following the procedures developed by Goldberg and Kiernan [11]. This group-based session features a PowerPoint presentation that includes information on the Group-Based and Self-Directed lifestyle programs, the research process, and the meaning of informed consent. It provides potential participants with an opportunity to discuss the pros and cons of participation with each other and decide whether or not the trial is right for them. These discussions include such considerations as current life priorities, time needed for lifestyle change, confidence in making changes at the current time,

and expected barriers. They are then asked to consider participation over the upcoming week, and then to call the site research staff if they continue to be interested in participating.

Step 3: Baseline Exam

The baseline exam has 3 components. If a prospective participant fails to attend a session or complete a required component, he/she is given one opportunity to reschedule or retry, except in special circumstances, as judged by the site Principal Investigator.

Component 1 (Visit A). The first in-person visit includes obtaining informed consent, demographic information, and assessment of eligibility criteria by questionnaire, medication questionnaire, and instructions for the Run-In period.

Component 2 (Run-In Period). A Run-In period was developed to identify those who are willing and able to engage in a selected set of activities required by the Group-Based program. This Run-In includes successful completion of the accelerometer protocol (wear accelerometer for 7 consecutive days, 10 hours/day), food and beverage logs (record all food and beverage intake over an entire day for a minimum of 3 weekdays and 1 weekend day), and completion of a worksheet that outlines a logistical plan for how to access vegetables and a location for physical activity (completed at home or during Visit B).

Component 3 (Visit B). The second in-person visit includes a 12-hour fasting blood draw, physical measures, confirmation that the Run-In protocol was completed successfully, and completion of baseline questionnaires. Visit B occurs within 1 month of randomization. For Wave 4, components of the metabolic syndrome may be collected up to 3 months before randomization. At the conclusion of Visit B, prospective participants are asked about continued interest, informed of expected wait time until the start of the program, and entered into the recruitment waiting protocol where they are kept informed of likely dates of starting treatment in ongoing contacts every 2 weeks. If a prospective participant is ineligible based on metabolic syndrome criteria or needs medical clearance for trial participation, the research assistant will follow-up with a phone call.

Randomization

Randomization is conducted by the RCU in permuted blocks and stratified by site. Since 1 of the arms in the trial is a group-based treatment of 15 participants/group, randomization will occur when 30 prospective participants are confirmed to be eligible and interested. Treatment should begin immediately after randomization. Upon randomization, participants are informed of the arm to which they were randomized by a mailed letter, an email, and a follow-up phone call from the respective site interventionist.

As randomization progresses, key population descriptors are compared in the trial arms to ensure that randomization is successfully balancing arms on key baseline characteristics. If any key covariate, such as age, race, gender, employment status, weight, or MetS component, differs by treatment arm, additional stratification in the randomization scheme is considered.

INTERVENTIONS

Highly motivated participants are recruited to help determine the better and more cost-effective way to encourage sustained lifestyle change to reverse the MetS.

The two arms are similar in the evidence-based content they provide, access to the website, and the use of a Fitbit to self-monitor physical activity. In both arms, lab results are sent to the participant and, with participant permission, to the primary care provider with encouragement to discuss the results. The arms differ in mode of delivery and intensity of contact. The Group-Based arm features face-to-face group contacts and a common protocol for accomplishing sustained behavior change. The Self-Directed arm features individual initiative and an individually-determined implementation schedule for achieving sustained behavior change. Within a context of highly-motivated patients, it is currently unclear which of these treatment approaches is better.

The Group-Based Arm

The Group-Based lifestyle program is the former Eat Love Move (ELM) lifestyle program, renamed in keeping with equipoise. It aims to achieve sustained change in lifestyle through the development of 4 habits that become automatic in daily life. The hypothesized pathway guiding this program is shown in **Figure 3** below. This is a 3-component treatment, targeting simultaneously diet, physical activity, and mindful awareness. Each component is translated into an ELM Leaf which is an easy-to-remember phrase representing each of the 4 habits. Each habit has a quantified implementation (enactment) target (in red), which is monitored by interventionists on a weekly basis. Success in achieving automaticity in each of these 4 habits is the behavioral mediator. The hypothesis is that if these 4 habits become automatic parts of daily life, they will translate into a clinically significant improvement in the behavioral risk factor targets of vegetable intake, physical activity, and mindful awareness. If clinically significant improvement in these health behaviors is achieved, the hypothesis is that this will translate into clinically significant weight loss (the biological mediator) and sustained remission of the MetS (the primary outcome).

Figure 3. Hypothesized pathway

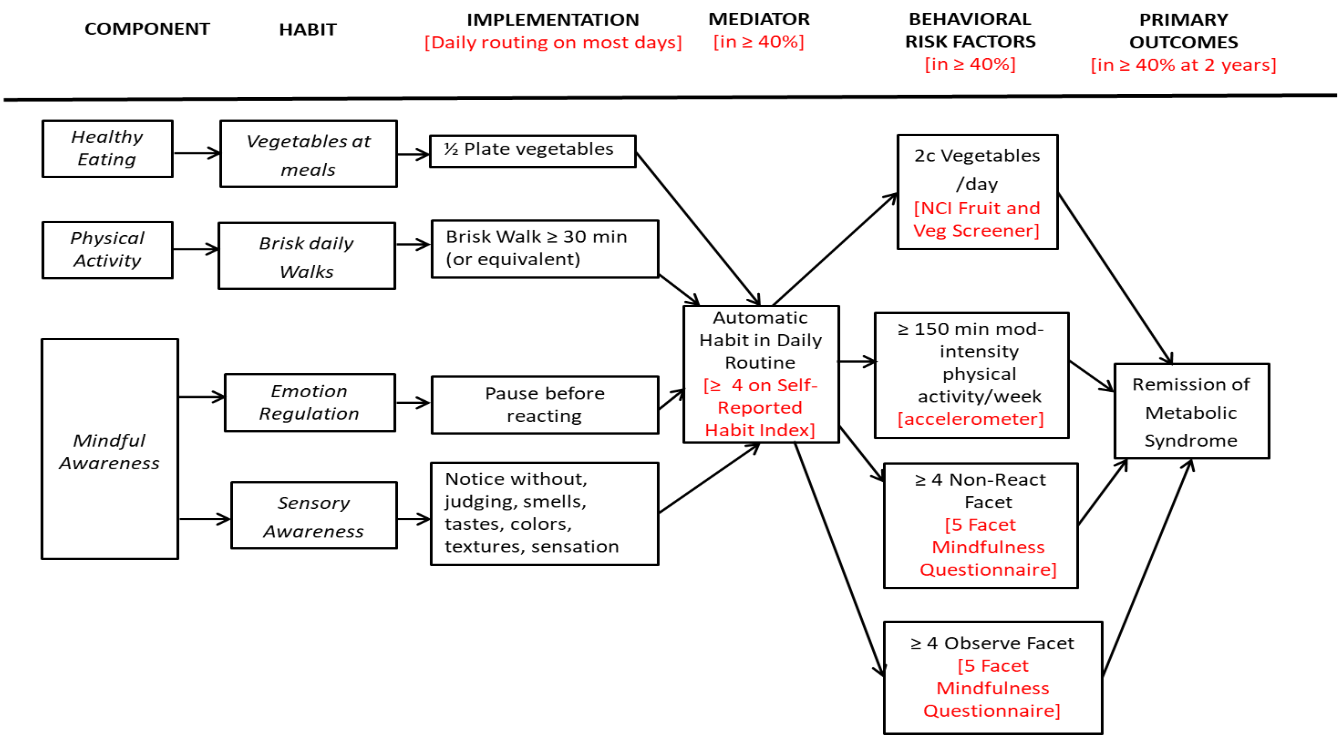

The intervention team at each site consists of a health psychologist and a registered dietitian, supported by a health coach (e.g. lay person who has achieved success with lifestyle modification). At baseline and 6 months, each participant meets with the dietitian for individual consultation. Together the psychologist and dietitian co-facilitate group meetings of approximately 15 participants. There is an intensive phase where meetings are held weekly for 3 months and bi-weekly for 3 months. The first 3 months is aimed at initiating change, focusing primarily on food intake, eating behavior, and weight. The last 3 months is aimed at maintenance, focusing on physical activity and a variety of other strategies that enhance maintenance. A maintenance phase follows the intensive phase where participants meet on a monthly basis to check in, report on challenges and successes, and discuss a new topic of their choice.

The format for all of the sessions in the intensive phase is an ecologically valid set of activities within which old habits emerge and new habits can be explored. Participants walk together, prepare a vegetable dish together, and eat while participating in an experiential exercise. The duration of each session is 90 minutes: physical activity (20 min), cooking demonstration (20 min), and discussion (50 min).

The maintenance phase (months 7 to 24 after randomization) consists of 60-minute monthly meetings, with participant input regarding the session topic. Participants are encouraged to broaden their health network by opening maintenance sessions to members of other ELM groups at the site. Meetings are available twice a month, one in-person and one virtual, for the duration of the follow-up to minimize non-attendance due to competing activities. Sessions may be offered one time per month when only one cohort is active. The health psychologist and dietitian alternate in supporting the meetings.

The Self-Directed Arm

The Self-Directed lifestyle program is intended to represent an enhanced version of what is currently available in clinical practice for patients who express an interest in using lifestyle change to manage their MetS. The current state-of-the-art in primary care for lifestyle management is the provision of evidence-based health education, featuring clear guidelines and behavioral targets, endorsed by the American Heart Association, American Diabetes Association, United States Department of Agriculture, and the Academy of Nutrition and Dietetics. Educational tip sheets are sent to participants on a monthly basis by their preferred method. An additional enhancement is provided by giving participants access to the ELM website that contains educational materials and other trial-related information.

The Self-Directed arm has been designed to serve as a clinically relevant comparator. It is intended to be the optimum status quo in medicine and technology, to which the efficacy and the cost-effectiveness of the Group-Based intervention can be compared. It has not been designed to control for attention, content, format, or other potential treatment mechanisms.

Each site has a Self-Directed Coordinator who meets with each participant post-randomization to introduce them to the components of the Self-Directed program and train them on use of their Fitbit and the ELM website. The Self-Directed Coordinator is available, via telephone, to answer participant questions about the trial. The Coordinator calls the participants every 3 months to answer questions about the trial and to enhance protocol adherence. The Coordinator does not answer medical or behavioral questions about lifestyle change. Instead, the participant is referred back to the relevant educational sheet or the primary care physician to get such questions answered. The essential role of the Coordinator is to facilitate participant ability to adhere to the protocol and to prevent risk of drop-out.

Fidelity Monitoring

Group-Based Program Fidelity

Interventionists attend 3 days of training held by the Intervention Coordinating Unit at Rush University Medical Center in Chicago. Treatment fidelity, guided by the recommendations of the NIH Behavior Change Consortium [12], assesses 3 dimensions of fidelity: delivery, receipt, and enactment, all of which are tracked by the Research Coordinating Unit and overseen by the Intervention Coordinating Unit supervisors in weekly meetings. Delivery of the treatment is enhanced by the availability of video recordings of each group session illustrating key points. Selected video recordings are reviewed by Intervention Coordinating Unit supervisors, written and verbal feedback is provided, and additional training is conducted, as needed. Receipt of the treatment is assessed by participant attendance records and brief comprehension forms completed by participants after each session. Enactment of the treatment is monitored by reviewing physical activity data from the Fitbit and weekly participant self-reported engagement in the 4 ELM habits (e.g. eating ½ plate of vegetables on most days, taking 7,500 steps on most days, pausing before responding, and noticing thoughts, bodily sensations, smells, colors, and tastes).

Self-Directed Program Fidelity

The Self-Directed Coordinators at each site participate in a 4-hour WebEx training led by the Intervention Coordinating Unit and the Self-Directed Program Supervisors. During this training, the Self-Directed Program Manual is presented. The coordinators engage in role plays to learn how to best navigate participant questions. All contacts between the Self-Directed Coordinators and participants are tracked by the Research Coordinating Unit. The Intervention Coordinating Unit reviews monthly reports to ensure all one-on-one orientation sessions are completed within 3 weeks of randomization, mailings are sent in a timely manner, and quarterly phone calls are completed. The site Coordinators meet monthly with the Self-Directed Program Supervisors to address any problems, concerns, and possible drift from the protocol.

OUTCOMES ASSESSMENT

In-Person Follow-Up Visits

These visits are intended to document the impact of the treatments over time. Thus, it is important that the timing of the assessments align with pivotal dates associated with intervention delivery (e.g., completion of the 6-month intensive phase, half-way through the 18-month maintenance phase, at the end of the trial at 24 months).

The timing of in-person follow-up visits is presented in **Table 1.** This timing applies to all participants, regardless of the treatment arm to which they were assigned. The target date of first follow-up visit is one day after the date of the last meeting in the intensive phase of the Group-Based arm. The second and third follow-up visits are 9 and

Table 1. Timing of In-Person Follow-Up Visits

Visit #	Schedule
First (Post-Intensive Intervention – PII)	Date of last group session of intensive phase (session 19)
Second (Mid-Maintenance – MM)	~9 months after intensive phase
Third (End of Treatment – EoT)	~18 months after intensive phase

18 months post-intensive phase, respectively. All follow-up visits have a 9-week window for completion that opens on the target date. 15-month assessment data can be collected after the window is closed up to the 21 month mark. Because of this, the 15-month window can overlap with the 18-month call. RA's can use discretion in regard to skipping the 18- month call if they are actively collecting in-person data for the 15 month assessment. The 24- month window is still 9 weeks. However, late data will be accepted until the day prior to trial closeout.

The essential goals of these visits are to collect follow-up data, update/confirm contact information, monitor adverse events, and promote retention. These contacts also provide the opportunity to sustain a professional and caring relationship with the participant throughout the 2-year trial. All visits are conducted by the same staff member, whenever possible, to cultivate a relationship that can aid in retention.

Telephone Contacts

The main purpose of the telephone contacts is to ascertain vital status, obtain information on health care utilization and co-interventions, and update contact information. These contacts occur at 3 months from randomization (for the first telephone contact) and then every 3 months after the date of the last group session. There is a total of 5 telephone contacts. This schedule is the same for all participants randomized at the same time, regardless of the arm to which they were assigned. The window for completion of telephone contacts is 2 weeks before and after the target date.

Assessments

Table 2 presents the assessments completed at each trial visit.

Change of Status

The trial status of a participant refers to their level of participation in the trial. A *Participant Status Form* is completed whenever a status change occurs. All efforts are made to collect data on the primary outcome (MetS at 2 years), at the very least.

Lost-to-Follow-Up. A participant is considered “lost-to-follow-up” after missing two consecutive trial contacts without responding to messages, texts, or other forms of communication. Staff exhaust all avenues to locate the participant, including telephoning at least 10 times on different days, at varying times of day, contacting secondary contacts and the last medical provider on record, and checking online to identify new contact information. Failure of these attempts triggers a registered letter sent to the participant's most recent address, to which non-response confirms the status as “lost-to-follow-up.”

Withdrawal from the Intervention. This status applies to a participant who no longer wants to participate in any intervention activities but is willing to continue completing follow-up visits.

Withdrawal from the Trial. This status applies to a participant who is no longer willing to participate in any part of the ELM trial, including group meetings, phone calls, or follow-up visits. However, the participant may allow the trial to use lab and medical data routinely collected by his/her provider.

Table 2. Assessments by trial visit and assessor

**Assessment**	**Frequency** B=Baseline, PII = Post-Intensive Intervention, MM = Mid-Maintenance, EoT = End-of-Treatment	**Assessor** RA = Research Assistant PPT = Participant
Accelerometry	B, PII, MM, EoT	RA
Vital Status, Adverse Events, Co-Interventions	3, PII, 9, 12, MM, 18, 21, EoT	RA
Contact Info	B,3, PII, 9, 12, MM, 18, 21, EoT	RA
Five-Facet Mindfulness Questionnaire	B, PII, MM, EoT	RA
Eating Competence Questionnaire	B, PII, MM, EoT	RA
Demographics	B, PII, MM, EoT	RA
Labs (Lipid Panel, Glucose and A1c)	B, PII, MM, EoT	RA
Medical History	B, PII, MM, EoT	RA
NCI's Fruit and Vegetable Screener	B, PII, MM, EoT	RA
Food Log and Logistic Plan	B	RA
Perceived Stress Scale	B, PII, MM, EoT	RA
Depression: Patient Health Questionnaire (PHQ-8)	B, PII, MM, EoT	RA
Physical Measures (BP, HR, Waist, Weight, Height)	B, PII, MM, EoT	RA
Automatic Habits: Self-Report Habit Index	B, PII, MM, EoT	RA
Social Support for Diet and Exercise	B, PII, MM, EoT	RA
Sugar-Sweetened Beverage Intake	B, PII, MM, EoT	RA
Visit log	B, PII, MM, EoT	RA
Quality of Life: Short Form Health Survey (SF-36)	B, PII, MM, EoT	RA
Contacts with Health Network	B, PII, MM, EoT	RA
Prescription Medications	B, PII, MM, EoT	RA
MacArthur Ladder of Subjective Social Standing	B	PPT
USDA Food Security	B	RA
Treatment Credibility Questionnaire	B	PPT
Patient Preference for Each Treatment	B	RA
Program Satisfaction	EoT	PPT
Substance Use/Abuse - WHO ASSIST	B	RA
Montreal Cognitive Assessment	B	RA (if needed)

Administrative Withdrawal. This status applies to any participant who poses a safety risk for other participants or trial staff. The ELM Safety Committee makes this determination in collaboration with the local site PI. This status terminates all further contact with the participant.

Deceased. This status is assigned once confirmed by the Safety Committee after review of the adverse event.

RETENTION

Retention has been excellent (90-100%) in the past clinical trials administered by the coordinating center at Rush. However, to assume a conservative approach, the sample size is calculated assuming a 20% loss to follow-up at 24 months. Key strategies to enhance retention include: (1) patient-centered recruitment emphasizing consideration of both the pros and cons of participation, from the participant's perspective; (2) maintaining 2-3 alternative contacts for each participant at enrollment; (3) same staff member assesses outcomes and maintains regular contact with participant over time; (4) minimization of transportation burden by providing free parking and transportation vouchers; (5) reimbursement for assessment time; (6) reminders about appointments for scheduled intervention and follow-up visits; (7) birthday and holiday cards; (8) collaborative partnerships with providers in participating clinics to enhance their support for their patient's participation; and (9) negotiating with potential drop outs to encourage partial withdrawal rather than withdrawal of consent. To reduce differential drop out, all staff are trained and re-trained to ensure equipoise, enthusiasm for a conclusive answer to the question rather than a specific result, and timely response to participant inquiries.

Participants at retention risk, based upon non-attendance at group meetings or inability to contact during 3-month phone calls, triggers a retention protocol. Depending upon the unique situation, the appropriate person (Group-Based interventionist, Self-Directed Coordinator, Outcomes Assessor) makes every effort to contact the participant for the purpose of understanding their perspective and any life situation that may have triggered the retention risk. For participants who are firm about decisions to terminate contact with the trial, every effort is made to keep the door open for a change in this decision at a later time. This is done by requesting the participant's permission to call at a later time to check it, the timing of such a call to be mutually agreed upon.

DATA FLOW

The trial has two main data flows. One is for recruitment and outcome assessment and the other is for intervention support. Data are collected using a variety of electronic systems including:

- **Snap Surveys.** Advanced software that allows for the customization of online data capture forms.
- **SharePoint.** A secure platform that houses the ELM website. SharePoint allows for sharing of data, documents, and publically accessible information.
- **CentrePoint.** A secure platform that transfers accelerometry to the Research Coordinating Unit (RCU).
- **Quanum Portal.** A secure platform that transfers participant lab results directly to the RCU.

Reports provided to investigators feature site-specific aggregated data on recruitment, outcomes, and intervention quality. Research Assistants and interventionists have access to the ELM website but are limited to viewing only data pertinent to their site and job responsibilities.

Recruitment and Outcome Assessments

Outcome collection begins at recruitment and continues in 3-month intervals over the 2-year follow-up period. The specific outcome variables collected can be found in the **Outcomes Assessment** section. The RCU provides recruitment reports and data tracking tables, via the ELM website, to help Research Assistants monitor recruitment and submission of outcome measures. **Figure 4** presents the flow of outcomes data.

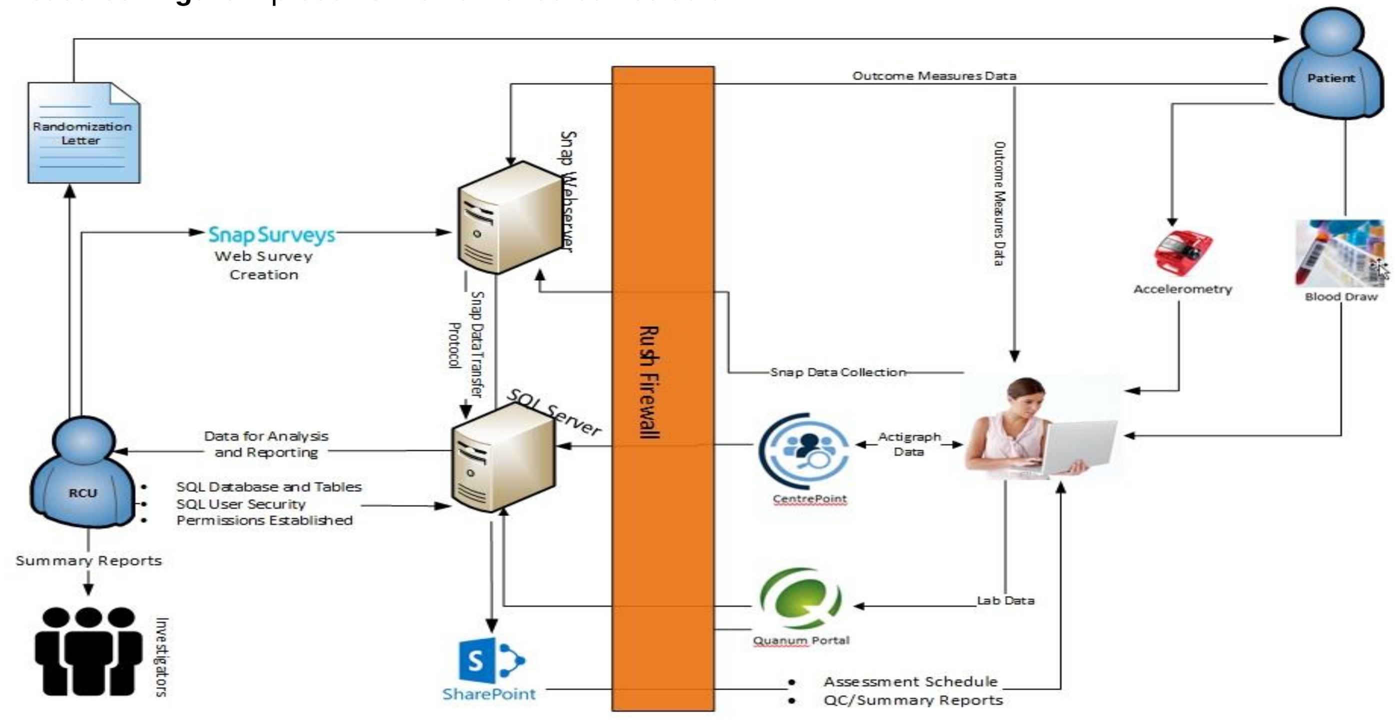

Figure 4. Elm Trial Outcomes Data Flow

Intervention Assessments

The RCU provides delivery support for specific intervention components and reports on the quality of intervention delivery.

In the Group-Based arm, the RCU supports the intervention by delivering automatic text messages that are tailored to content presented in the prior week and sent on a pre-existing schedule. At selected group meetings, the RCU prepares personalized feedback reports for each participant. The RCU creates reports on intervention progress assessed as class attendance, weight loss compared to the goal of > 5% loss of baseline body weight, and step count compared to the goal of 7,500 steps on most days. The RCU also creates reports on 3 treatment fidelity measures:

- **Delivery.** Documentation of delivery of specific intervention components.
- **Receipt**. Participant self-reports of acquisition and understanding of specific group content.
- **Enactment**. Participant self-reports of daily vegetable intake, mindfulness habits, and old habit recognition.

These reports are uploaded weekly by the RCU to relevant receivers via the ELM website, including interventionists, participants, investigators, and Intervention Coordinating Unit supervisors.

In the Self-Directed arm, data collected by the Self-Directed Coordinator includes completed telephone calls and frequency of participant-initiated contacts. These data are sent to the RCU and displayed quarterly in aggregate form on the ELM website for review by the Self-Directed Coordinator, investigators, and the ICU supervisors.

Participants in both arms are able to view their own data and track their progress in step counts and weight change. The RCU also tracks frequency of daily log-ons to the ELM website by participants in both arms. **Figure 5** presents the flow of intervention data.

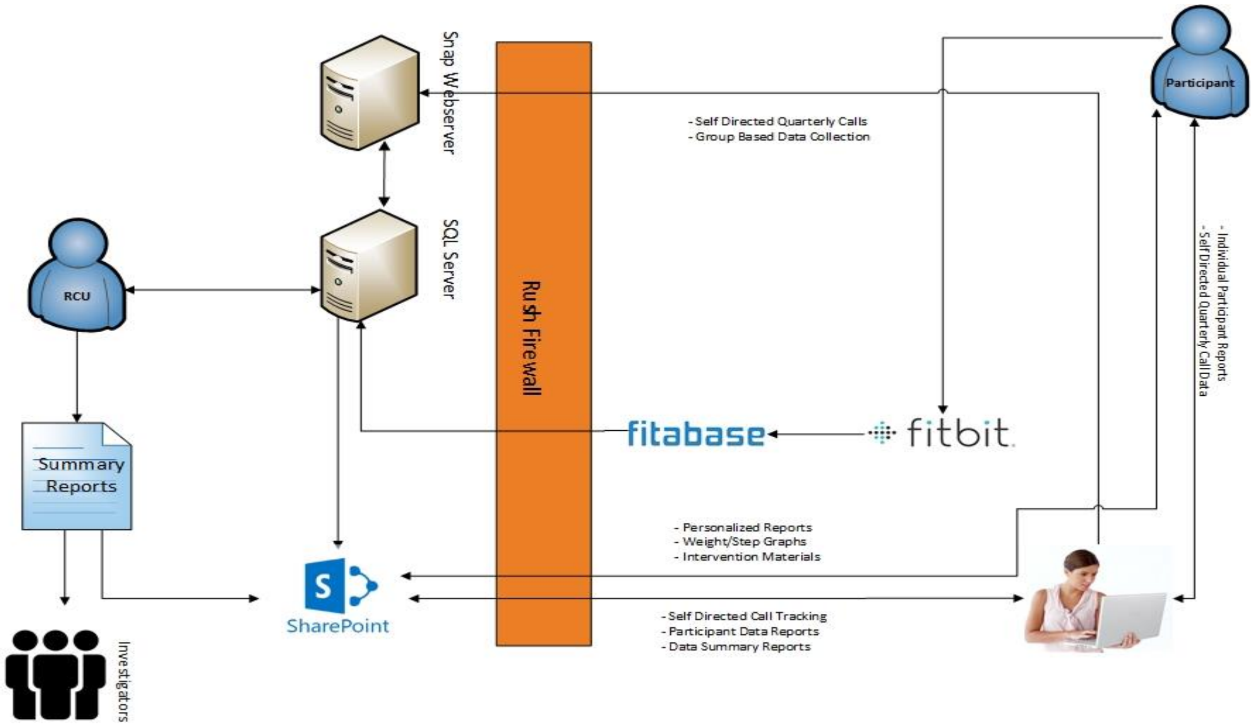

Figure 5. Intervention Data Flow

STATISTICAL CONSIDERATIONS

Sample Size

The primary outcome of the trial is remission of MetS at 24 months. MetS remission (meeting criteria for <3 MetS components) is clinically relevant and the most common endpoint in MetS trials. Sample size was based upon the assumptions that the chi-square test will be used to compare proportions of participants who remit MetS at 24 months by trial arm, with an overall significance level of 0.05, and power of 90%. This requires a sample size of 117 participants per group, or a total sample size of 234.

Consideration must be given to the fact that the proposed trial employs a “partially clustered” design where clustering is present in only 1 of the 2 arms. The Group-Based Program is

delivered to participants in groups, or clusters, of approximately 15 participants. Connection and interactivity within each of these "experimentally-induced" clusters is a key component. The intraclass correlation coefficient (ICC) measures the extent to which participant responses are influenced by the cluster to which they belong. Since this clustering feature is present in only one of the two arms, its effect is muted. Results from our previous studies are consistent with those of others [13-14] indicating that the ICC could reasonably range from 0.01 to 0.20. We conservatively assume an ICC of 0.15 and adjust the total sample size required to achieve 90% power to 286 per arm or a total sample size of 572. We adjust upward to 300 participants/arm, a total sample size of 600 participants, 120 participants at each of 5 clinical sites, and 8 intervention clusters per site.

Analysis Plan

Analyses will be performed using an intent-to-treat approach that includes all patients as randomized, regardless of treatment dose. All analyses will use two-sided tests conducted at $\alpha=0.05$. Basic summary statistics will be based upon cluster-adjusted chi-square tests and t-tests. If a distribution is non-normal, cluster-adjusted Wilcoxon rank sum tests will be used. Supplemental analyses will include mixed effects regression models to account for cluster effects and control for important covariates. The impact of missing data will be evaluated using sensitivity analyses. If data are missing at random, multiple imputation will be employed. If data are not missing at random, an appropriate statistical model will be employed (e.g. a single imputation strategy with sensitivity analyses using pattern mixture models).

The primary hypothesis is achievement of a clinically significant MetS remission at 24 months in $\geq$ 40% of the participants in the Group-Based program and $\leq$ 20% of the participants in the Self-Directed program. Achieved rates will be observed, relative to these benchmarks, without statistical support. The primary statistical analysis will compare MetS remission at 24 months between the trial arms using a cluster-adjusted chi-square test. A secondary analysis will compare these rates using a mixed effects logistic regression model examining treatment over time, a treatment*time interaction, and adjustments for pre-specified covariates: age, gender, race/ethnicity, socioeconomic status, geographic site, BMI, number of co-morbidities, and number of MetS components. Random effects (e.g., random intercept, time, intervention cluster) will be included in the model only if they significantly improve model fit as determined by the likelihood ratio test.

Behavioral treatment targets will be compared by arm, without statistical support, as the proportion of participants achieving clinically significant goals (as presented in the hypothesized pathway in **Figure 3**), based upon the pre-determined benchmarks. Statistical support will be provided by a cluster-adjusted chi-square test. All other secondary and exploratory outcomes will be compared using the relevant cluster-adjusted t-test or chi square test. These comparisons will be conducted using data from post-intensive intervention and end-of-treatment visits and trends over time will be examined. Supplementary analyses will use generalized linear mixed effects modeling, with a behavioral treatment target or weight as the outcome, treatment group as the primary predictor, random effects to account for time, site, and cluster, and selected covariates.

Exploratory analyses examine the efficacy to treatments within important population subgroups. These comparisons will be presented by forest plots. Intervention fidelity within each treatment arm is monitored on an ongoing basis by the Intervention Coordinating Unit, with remediation delivered promptly when problems are identified. Observed fidelity data will be compared to the clinically significant goal of at least 80% adherence to key components for the overall trial and stratified by geographic site.

The health economic evaluation features the incremental cost effectiveness ratio (ICER) for the 2 trial arms, where the ICER = $(C_1 – C_0)/(E_1 – E_0)$, and C is intervention cost, E is effectiveness at remission of MetS, subscript 1 denotes the Group-Based arm, and subscript 0 denotes Self-Directed arm. The ratio yields the cost differential for MetS remission in the 2 trial arms. In addition, the ICER will be calculated for each Quality-Adjusted Life Year (QALY) gained. The analysis will be conducted from the commercial payer, public payer, and societal perspectives. Sensitivity analyses will be conducted to account for the potential influence of plausible changes in cost assumptions.

The projected benefits of disseminating the Group-Based Program will be modeled by combining the observed rate of MetS remission in the trial with published national estimates of cardiometabolic disease risk and added health care costs associated with MetS. Projections will focus on the number of potential cases of type 2 diabetes, coronary heart disease, and stroke averted, and potential healthcare savings, in the overall adult population, Medicare population (65+), adults on Medicaid, and privately insured adults.

TRIAL ORGANIZATION

Figure 6 presents the structure of this multi-site trial. Leadership is provided by an eight-member Steering Committee comprised of the Principal Investigators of all 5 sites, the Trial Chair, the Research Coordinating Unit Director, and the Intervention Coordinating Unit Director. Decision-making uses consensus as a primary strategy. Votes on key questions are carried by a majority. Intervention training and fidelity is overseen by the Intervention Coordinating Unit. Quality of Data transmittal is overseen by the Research Coordinating Unit. Scientific Committees guide all scientific aspects of the trial. An independent Data and Safety Monitoring Board approves the trial protocol, monitors data quality and safety, and monitors outcomes by trial arm. It makes recommendations to the investigators and the funder by providing independent reports simultaneously.

SAFETY MONITORING

This trial presents low risk to participants given the nature of the lifestyle interventions and the trial measurements collected. Participants are thoroughly screened prior to enrollment to ensure their ability to participate in lifestyle interventions. Nonetheless, several types of unanticipated problems and adverse events may occur and will be monitored. All potential events reported by a participant or staff member are documented using the *Adverse Events Form.* This form is submitted to the Research Coordinating Unit (RCU), which will then submit it to the Site Physician within a predetermined timeline. The Site Physician adjudicates the event following guidelines and reports back to the RCU. The RCU sends the completed adjudication form to the Chair of the Safety and Ethics Committee and Trial Chair, as described below. Summary reports are provided to the Rush IRB and the Data and Safety Monitoring Board. The Chair of the Safety and Ethics Committee can alert the Data Safety and Monitoring Board at any other time at his/her discretion.

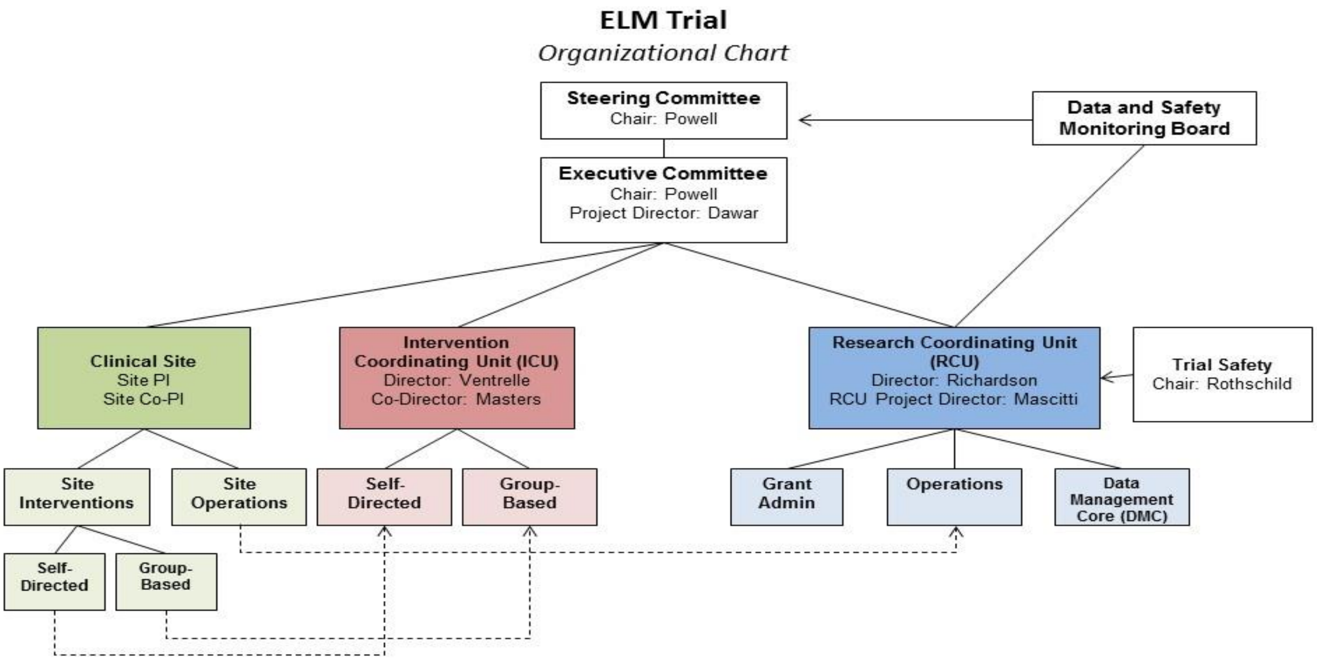

Figure 6. ELM trial organization

Adverse Events

The following procedures occur, dependent upon determination of the event.

- **Adverse Events** and **Serious Adverse Events** that are <u>not</u> Unanticipated Problems are submitted to the central IRB in the annual Continuing Review application. These events are officially reported to the Steering Committee at its monthly meeting and semi-annually to the DSMB.

- **Unanticipated Problems** are submitted to the central IRB within 1 week of the Safety & Ethics Committee's review. The Steering Committee is to be notified promptly, and in no case longer than one month from the Committee's review. The DSMB is to be notified quarterly.

A summary of this reporting process is presented in **Figure 7.**

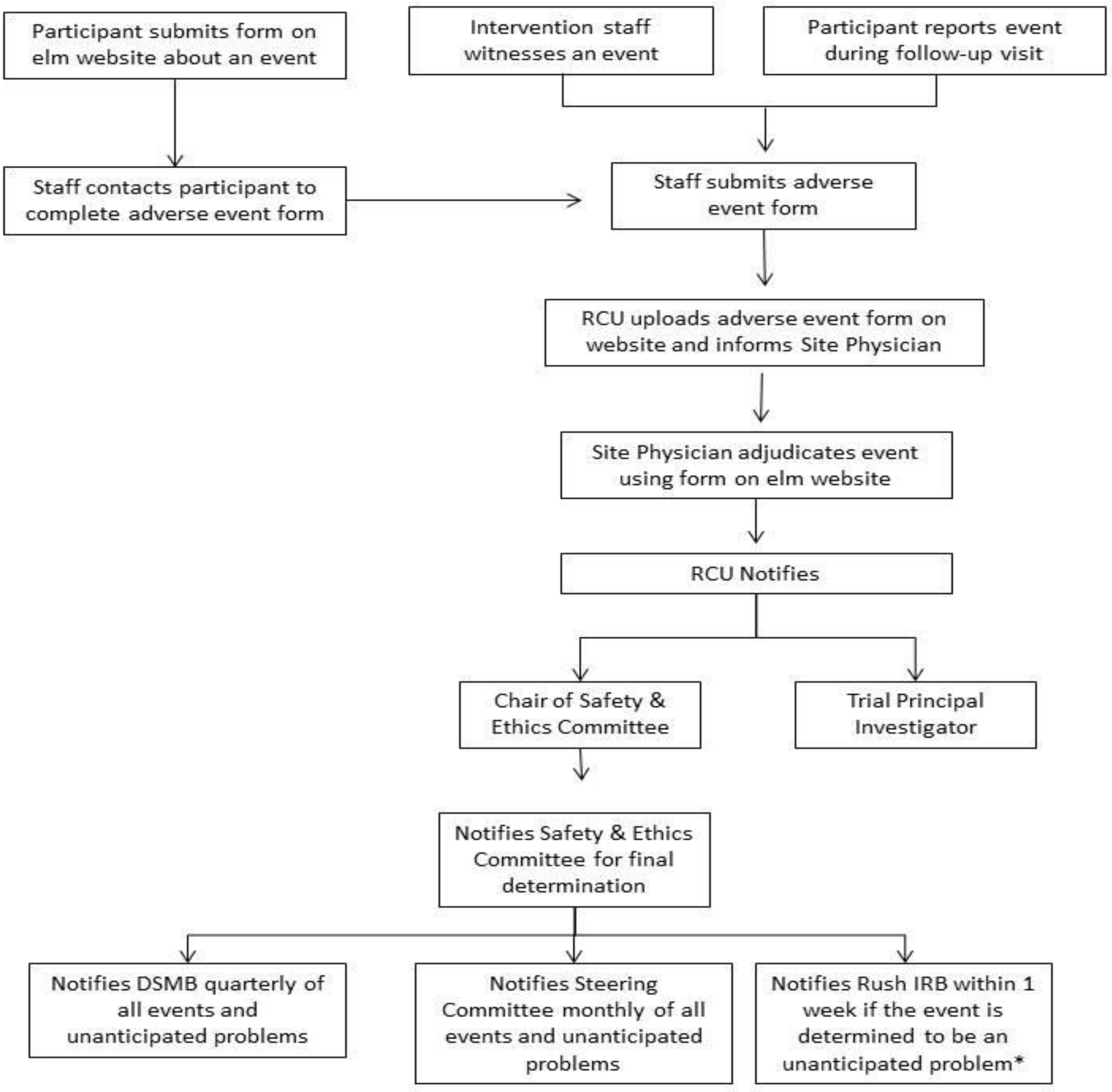

*An *Unanticipated Problem* is an incident, experience, or outcome that meets all of the following criteria:

- The nature, severity, or frequency is unexpected
- It is related or possibly related to participation in the research.
- It suggests the research may place subjects at greater risk of harm than previously recognized.

Figure 7: Process of Unanticipated Problems and Adverse Event Reporting

Critical Alert Values

Immediate risk is posed, and actions are needed, for the following problems.

Blood Pressure Above 160/100. Should this event occur, the research assistant contacts the participant's provider or designee to obtain a recommendation for patient care, prior to the participant leaving. If the participant does not want their primary care physician to be contacted, then the research assistant contacts the Site Physician. If a provider cannot be reached within one hour or before the participant plans to leave, the safety protocol is as follows:

Blood Pressure between 160/100 and 180/110 and <u>no</u> symptoms. Participant is told that their blood pressure is elevated, and they need to see their doctor as soon as possible for repeat testing and evaluation.

Blood Pressure between 160/100 and 180/110 <u>with</u> symptoms, such as (but not limited to) chest pain, shortness of breath, blurred vision, or headache. Participant is urged to go to the nearest Emergency Department.

Blood Pressure higher than 180/110. Participant is urged to go to the nearest Emergency Department.

Glucose < 40 or ≥500; Triglyceride > 1,000. All lab results are reviewed by the Site Physician prior to being sent to a participant. If the glucose alert occurs, the Site Physician contacts the participant or his/her provider within 24 hours. If the triglyceride alert occurs, the Site Physician notifies the participant by phone and advises him/her to contact his/her primary care provider.

REFERENCES

1. Alberti KG, Eckel RH, Grundy SM, Zimmet PZ, Cleeman JI, Donato KA, Fruchart JC, James WP, Loria CM, Smith SCJ, International Diabetes Federation Task Force on Epidemiology and Prevention, National Heart, Lung, and Blood Institute, American Heart Association, World Heart Federation, International Atherosclerosis Society, the International Association for the Study of Obesity (2009) Harmonizing the metabolic syndrome: a joint interim statement of the International Diabetes Federation Task Force on Epidemiology and Prevention; National Heart, Lung, and Blood Institute; American Heart Association; World Heart Federation; International Atherosclerosis Society; the International Association for the Study of Obesity. Circ 120:1640-1645.
2. Go AS, Mozaffarian D, Roger VL (2014) Heart disease and stroke statistics - 2014 update: a report from the American Heart Association. Circ 129:e28-e292.
3. Grundy SM (2012) Pre-diabetes, metabolic syndrome, and cardiovascular risk. J Am Coll Cardiol 59:635–643
4. Hunt SA, Abraham WT, Chin MH, Feldman AM, Francis GS, Ganiats TG, Jessup M, Konstam MA, Mancini DM, Michl K, Oates JA, Rahko PS, Silver MA, Stevenson LW, Yancy CW (2009) 2009 focused update incorporated into the ACC/AHA 2005 Guidelines for the Diagnosis and Management of Heart Failure in Adults: a report of the American College of Cardiology Foundation/American Heart Association Task Force on Practice Guidelines: developed in collaboration with the International Society for Heart and Lung Transplantation. Circ 119:e391-e4791.
5. Moore JX, Chaudhury N, Akinyemiju T (2017) Metabolic syndrome by race/ethnicity and sex in the United States, National Health and Nutrition Examination Survey, 1988-2012. Prev Chronic Dis 14:160287.DOI:http://dx.doi.org/160210.165888/pcd160214.160287
6. Boudreau DM, Malone DC, Raebel MA, Fishman PA, Nichols GA, Feldstein AC, Boscoe AN, Ben-Joseph RH, Magid DJ, and Okamoto LJ (2009) Health care utilization and costs by metabolic syndrome risk factors. Metab Syndr Relat Disord 7:305-314.
7. Orchard TJ, Temprosa M, Goldberg R, Haffner S, Ratner R, Marcovina S, Fowler S, the Diabetes Prevention Program Research Group (2005) The effect of metformin and intensive lifestyle intervention on the metabolic syndrome: the Diabetes Prevention Program randomized trial. Ann Intern Med 142:611-619.
8. Rosmond R (2005) Role of stress in the pathogenesis of the metabolic syndrome. Psychoneuroendocrinology 30:1-10.
9. Bergmann N, Gyntelberg F, Faber J (2014) The appraisal of chronic stress and the development of the metabolic syndrome: a systematic review of prospective cohort studies. Endocr Connections 3:R55-R80.
10. Powell LH, Appelhans BM, Ventrelle J, Karavolos K, March ML, Ong JC, Fitzpatrick SL, Normand P, Dawar R, Kazlauskaite R (2018) Development of a lifestyle intervention for the metabolic syndrome: discovery through proof-of-concept. Health Psych 37:929-939.
11. Goldberg JH, Kiernan M (2005) Innovative techniques to address retention in a behavioral weight-loss trial. Health Educ Res 20:493-447.
12. Bellg AJ, Borrelli B, Resnick B, Hecht J, Minicucci DS, Ory M, Ogedegbe G, Orwig D, Ernst D, Czajkowski S, Treatment Fidelity Workgroup of the NIH Behavior Change Consortium (2004) Enhancing treatment fidelity in health behavior change studies: best practices and recommendations from the NIH Behavior Change Consortium. Health Psych 23:443-451.

13. Campbell MK Fayers PM, Grimshaw JM (2005) Determinants of the intracluster correlation coefficient in cluster randomized trials: the case of implementation research. Clinical Trials 2:99–107.
14. Dziak JJ, Nahum-Shani I, Collins LM (2012) Multilevel factorial experiments for developing behavioral interventions: power, sample size, and resource considerations. Psychol Methods 17:153-175.
15. Hedges LV (2009) Adjusting a significance test for clustering in designs with two levels of nesting. J Educ Behav Stat 34:464–490.
