# Supplementary material for: Lifestyle Intervention for Sustained Remission of Metabolic Syndrome: A Randomized Clinical Trial
Source: JAMA Intern Med. 2025 Nov 9;186(1):67–77. doi: 10.1001/jamainternmed.2025.5900 (PMC12598583; doi:10.1001/jamainternmed.2025.5900)
Supplement: Supplement 2. — eMethods 1. Informed Consent: Slides for Information Session eMethods 2. Consent Form eMethods 3. Sensitivity Analyses eMethods 4. Data Sharing and Dissemination eMethods 5. Table 2 (Addition) Secondary Endpoint eFigure 1. Metabolic Syndrome Remission By Trial Arm and Time, including the 15-month assessment eFigure 2. Prespecified Baseline Subgroups: Odds of Metabolic Syndrome Remission At 6 and 24 Months [file jamainternmed-e255900-s002.pdf]

Supplemental Online Content

Powell LH, Berkley-Patton J, Drees BM, et al; for the ELM Trial Research Group. Lifestyle intervention for sustained remission of metabolic syndrome: a randomized clinical trial. *JAMA Intern Med.* Published online November 9, 2025. doi:10.1001/jamainternmed.2025.5900

eMethods 1. Informed Consent: Slides for Information Session

eMethods 2. Consent Form

eMethods 3. Sensitivity Analyses

eMethods 4. Data Sharing and Dissemination

eMethods 5. Table 2 (Addition) Secondary Endpoint

eFigure 1. Metabolic Syndrome Remission By Trial Arm and Time, including the 15-month assessment

eFigure 2. Prespecified Baseline Subgroups: Odds of Metabolic Syndrome Remission At 6 and 24 Months

This supplemental material has been provided by the authors to give readers additional information about their work.

© 2025 Powell LH et al. *JAMA Intern Medicine*.

eMethods 1. Informed Consent. Slides for Information Session

(Adapted from: Goldberg JH, Kiernan M. Innovative techniques to address retention in a behavioral weight-loss trial. *Health Educ Res* 2005;20:439-447. doi: 10.1093/her/cyg139.

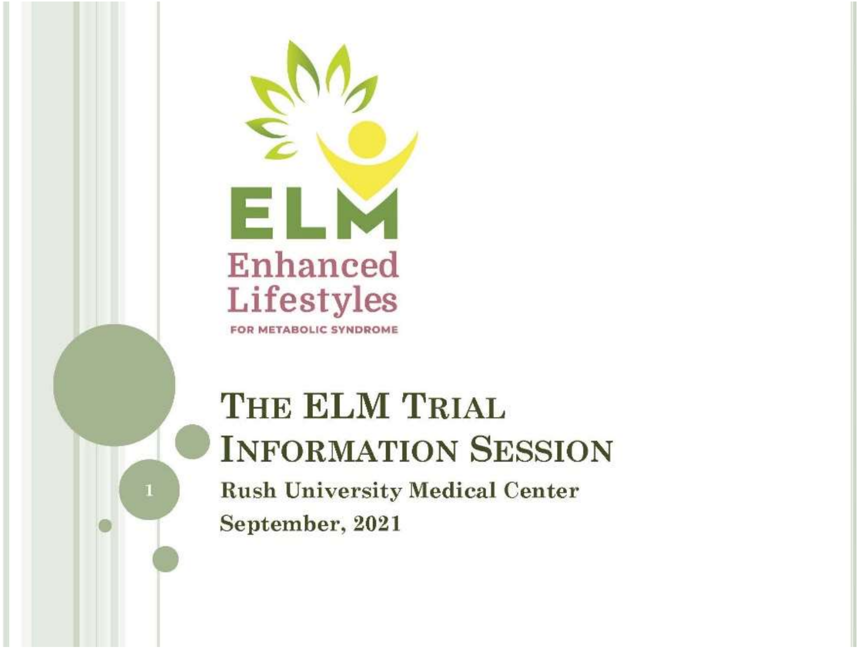

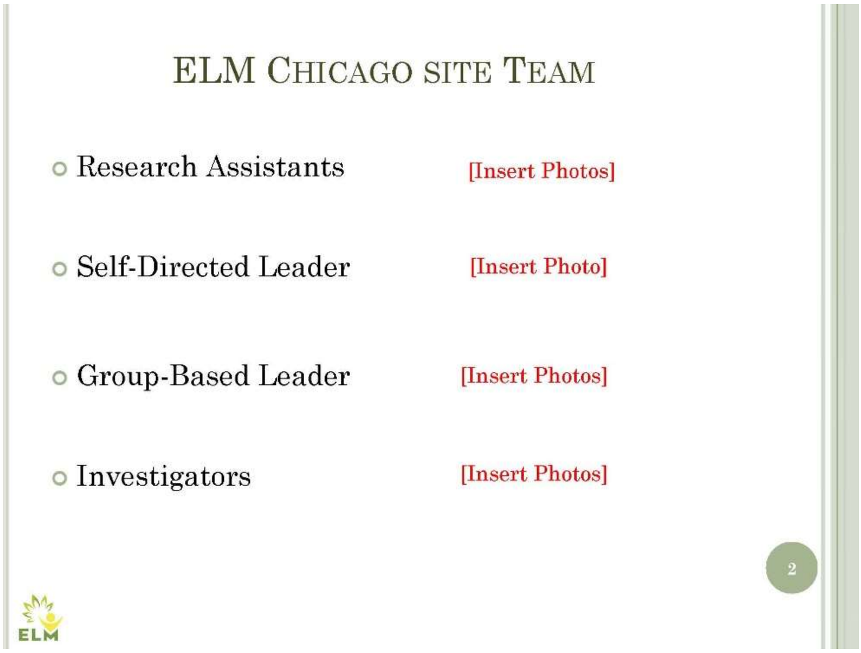

© 2025 Powell LH et al. *JAMA Intern Medicine*.

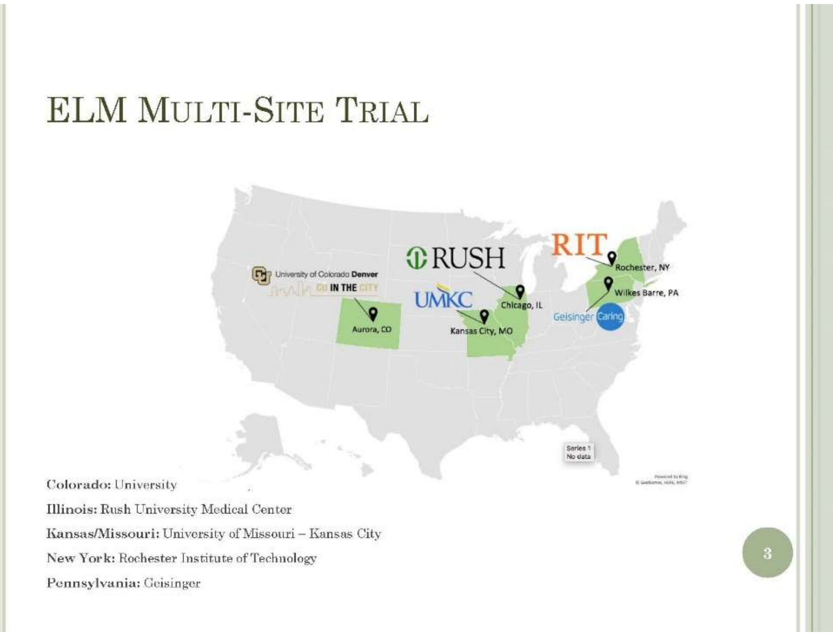

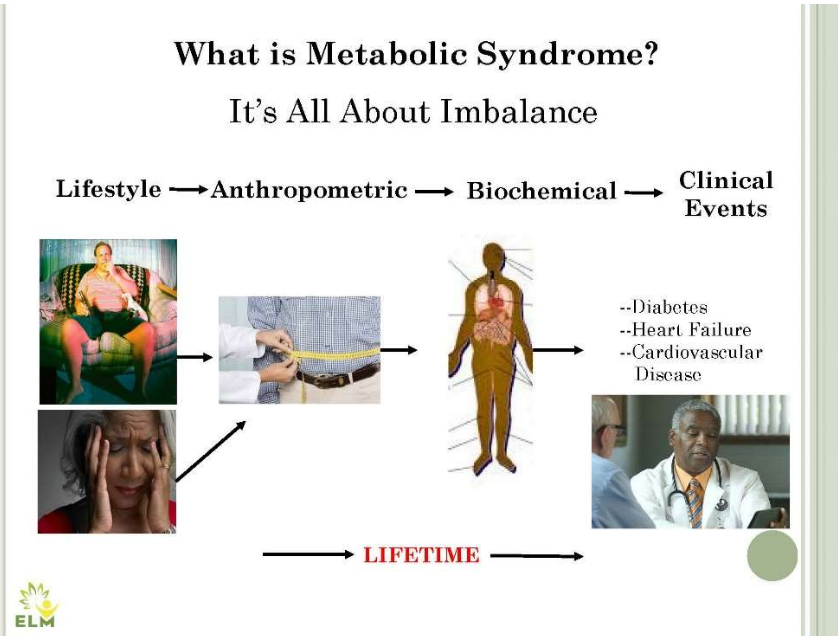

© 2025 Powell LH et al. *JAMA Intern Medicine*.

WHAT IS METABOLIC SYNDROME?

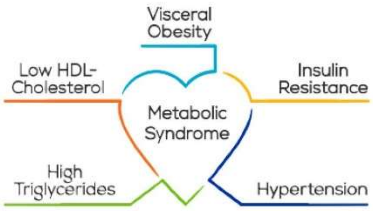

Having 3 or more risk factors:

- Waist circumference ≥ 40 inches (men) and ≥ 35 inches (women)
- Fasting blood glucose 100-125 mg/dL
- High blood pressure or on high blood pressure medication
- Triglycerides ≥150 mg/dL
- HDL cholesterol <40 mg/dL in men or <50 mg/dL in women

5

ELM

RESEARCH QUESTION

- Which lifestyle program is more effective in helping people reverse the Metabolic Syndrome and sustain it at 2 years?
- Self-Directed Program or Group-Based Program

6

ELM

© 2025 Powell LH et al. *JAMA Intern Medicine*.

GOAL

- Reversal of the metabolic syndrome

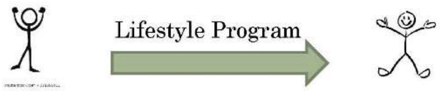

Assessment	Visit before Program
Blood Pressure	150/75 mmHg
Waist Circumference	45 inches
HDL "good cholesterol"	35 mg/dL
Triglycerides	120 mg/dL
Fasting Glucose	110 mg/dL
**Total Risk Factors**	**4**

Assessment	Visit at 2 Years
Blood Pressure	120/75 mmHg
Waist Circumference	43 inches
HDL "good cholesterol"	45 mg/dL
Triglycerides	120 mg/dL
Fasting Glucose	105 mg/dL
**Total Risk Factors**	**2**

ELM

7

HOW DO YOU GET PUT INTO A PROGRAM?

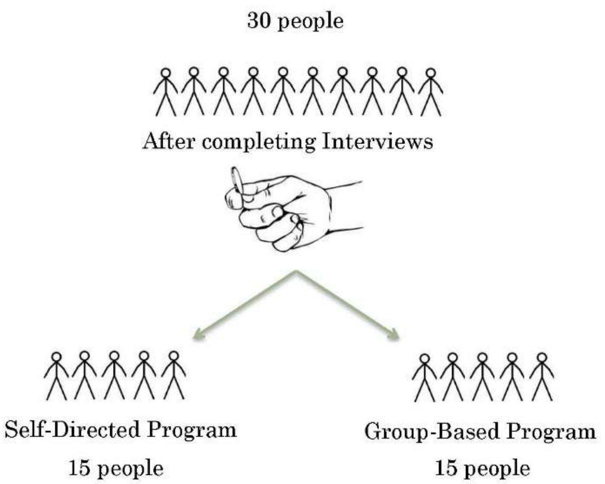

ELM

8

© 2025 Powell LH et al. *JAMA Intern Medicine*.

How do the programs compare?

Self-Directed Program	Group-Based Program
• Orientation session<br>• Educational mailings for 2y<br>• Independent learning	• Orientation session<br>• Group meetings for 2y<br>• Coaching
• Fitbit physical activity tracker	• Fitbit physical activity tracker
• ELM website access	• ELM website access
• ELM sends progress letter with lab results to primary care physician	• ELM sends progress letter with lab results to primary care physician

ELM

Self-Directed Program

Monthly Mailings

- About metabolic syndrome
- Know your numbers
- Nutrition
- Physical activity
- Stress reduction

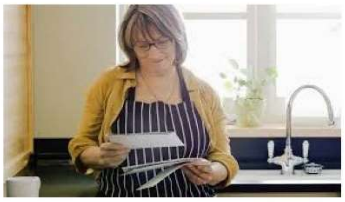

Participant Responsibilities

- Wear Fitbit daily
- Read educational materials and follow guidelines
- Attend all study visits

10

ELM

© 2025 Powell LH et al. *JAMA Intern Medicine*.

Time commitment Self-Directed Program

Activity	Min. session	Sessions/week	Total time/week
Review Mailings and Generate Action Plan	15 min	1 day	**15 min**
Physical Activity	30 min	5 days	**150 min**
Other Lifestyle Behaviors	30 min	7 days	**210 min**

About 6 hours/week

Adapted from: Kiernan M. Optimizing Retention for Randomized Clinical Trials: State of the Science, Innovation, and Implementation. NHLBI Summer Institute 2018.

Group-Based Program

Months 1-3:
- Weekly group meetings

Months 4-6:
- Twice monthly group meetings

Months 7-24:
- Monthly group meetings with graduates of past groups

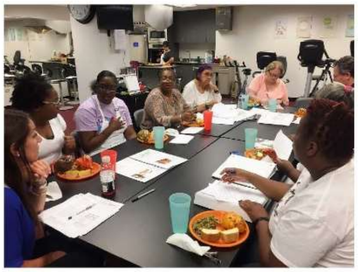

Participant Responsibilities:
- Wear Fitbit daily
- Attend at least 80% of group meetings
- Keep food log records for at least 3 months
- Devote $\geq$ 1 hour each day to lifestyle goals
- Attend all study visits

12

© 2025 Powell LH et al. *JAMA Intern Medicine*.

TIME COMMITMENT GROUP-BASED PROGRAM

Activity	Min. session	Sessions/week	Total time/week
Group time	90 minutes	1/week	**1.5 hrs**
Commute to/from sessions	40+ min	1/week	**40+ min**
Record keeping	15 min	7 days	**1.75 hrs**
Physical Activity	30 min	5 days	**2.5 hrs**
Other Lifestyle Behaviors	30 min	7 days	**3.5 hrs**

About 10 hours/wk during the first 3 months

Adapted from: Kiernan M. Optimizing Retention for Randomized Clinical Trials: State of the Science, Innovation, and Implementation. NHLBI Summer Institute 2018.

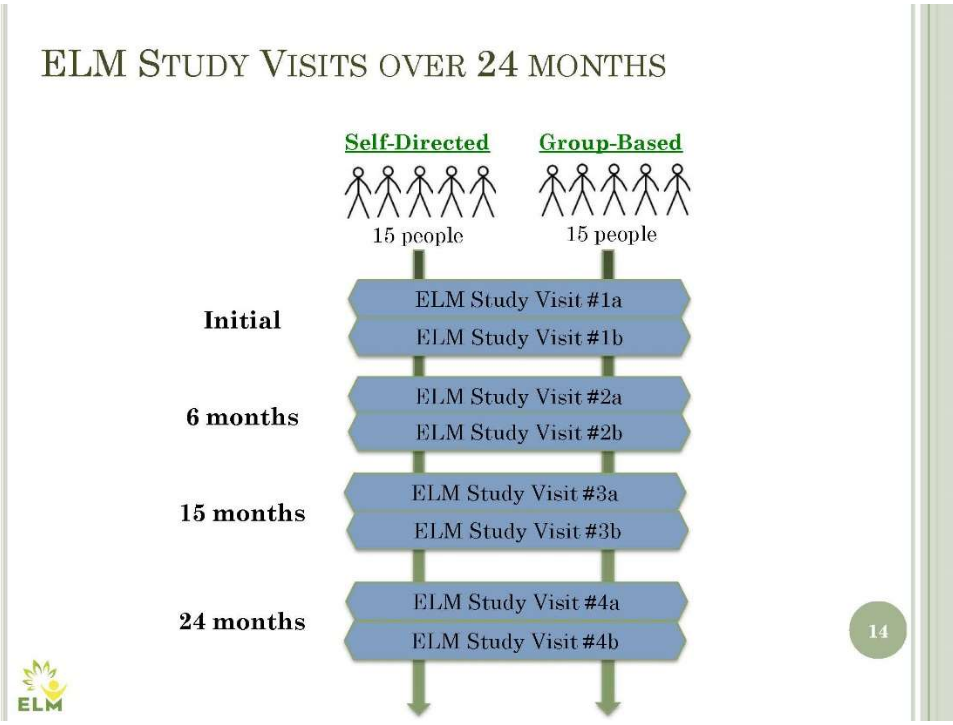

© 2025 Powell LH et al. *JAMA Intern Medicine*.

WHAT HAPPENS AT THE STUDY VISITS?

Visit 1a

- Consent
- Demographics
- Medications
- Physical measures
- Screening questionnaires
- Blood draw
- Home assignment:
  - Accelerometer
  - Food log
  - Plan of action

Do you have metabolic syndrome

?

1 week →

Visit 1b

- Behavioral questionnaires
- Home assignment reviewed by research assistant:
  - Wore accelerometer for 7 days
  - Completed food log
  - Responded to Logistical Plan questions

15

ELM

WHY IS IT SO IMPORTANT THAT EVERYONE COMES BACK TO THE STUDY VISITS?

If everyone comes back…

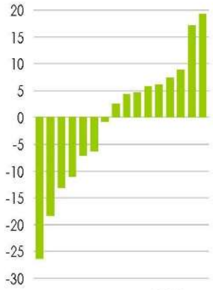

We want a true picture!

If only people who lost weight came back…

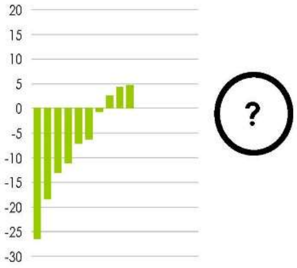

The program looks MORE successful than it really was

16

Adapted from: Kiernan M. Optimizing Retention for Randomized Clinical Trials: State of the Science, Innovation, and Implementation. NHLBI Summer Institute 2018.

© 2025 Powell LH et al. *JAMA Intern Medicine*.

BRAINSTORMING: Pros & Cons of Participating in the ELM Study

Is this study right for me?

Priority
When you consider the different priorities in your life right now, such as family, work, & community, how much of a priority is an intensive lifestyle program at this time?

0	1	2	3	4	5	6	7	8	9	10
Very Low										Very High

Time
This study will require a significant amount of time commitment, including purchasing and cooking healthy food, engaging in daily physical activity, and practicing new skills regularly. How much time can you realistically spend per day?

0 min	10 min	20 min	30 min	40 min	50 min	60 min	70 min	80 min	90 min	100 min

Confidence
How confident are you that you will be able to purchase and prepare vegetables and set up a regular physical activity routine?

0	1	2	3	4	5	6	7	8	9	10
Very Low										Very High

Barriers
How confident are you that you can exercise even when barriers come up (e.g., very bad weather, not fun or enjoyable, difficult to get to the exercise location, scheduling conflicts, lack of support from family or friends, or self-conscious about your appearance)?

0	1	2	3	4	5	6	7	8	9	10
Very Low										Very High

	Self-Directed	Group-Based
Pros		
Cons		

ELM

17

Self-Directed vs. Group-Based Program

White board

	Self-Directed	Group-Based
PROS		
CONS		

Adapted from: Goldberg J, Kiernan M. Innovative techniques to address retention in a behavioral weight-loss trial. *Health Educ Res.* 2005;20:439-447.

© 2025 Powell LH et al. *JAMA Intern Medicine*.

HOW DO I SIGN UP?

- **Contact Henry to schedule the initial screening visit**

[Insert Photo of Henry]

Information Sessions	Screening Visits	Program Assignment
September - December	September – December	January 2022

19

ELM

QUESTIONS?

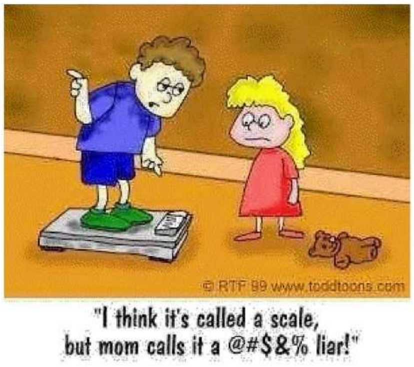

20

ELM

© 2025 Powell LH et al. *JAMA Intern Medicine*.

eMethods 2. Consent Form

ORA: 18092408-IRB01-CR02 Date IRB Approved: 2/15/2021 Expiration Date: 2/15/2022

RUSH

CONSENT/AUTHORIZATION FOR PARTICIPATION IN A RESEARCH STUDY

**Site Principal Investigator:**	BRADLEY APPELHANS, PHD
**Department:**	Preventive Medicine
**Address and Contact Information:**	1700 W. Van Buren Street<br>Suite 470<br>Chicago IL 60612<br>Phone: (312) 942-3477<br>Email: Brad_Appelhans@rush.edu
**Protocol Title:**	Enhanced Lifestyles for Metabolic Syndrome (ELM)
**Sponsor(s):**	William G. McGowan Charitable Fund
Sites:	Rush University Medical Center, University of Colorado at Denver, Geisinger Health System, University of Missouri-Kansas City, Rochester Institute of Technology
Version Date:	November 25, 2020

Name of Participant: ____

<u>Key Information</u>:

You are being invited to take part in a research study. Research studies answer important questions that might help change or improve the way we do things in the future.

This consent form will give you knowledge about the study to help you decide whether you want to be in the study. Please read this form, and ask any questions you have, before agreeing to be in the study.

Details about the things we will do and what we will ask you to do are shown on this document. You will have time to think about everything before you agree to be in the study.

The purpose of this study is to compare two lifestyle programs designed to reverse the metabolic syndrome (MetS), which is described later in the *"Detailed Information"* section. You will be asked to be in a lifestyle program designed to help people learn how to eat healthy, lower stress, and move more.

If you agree to be in this study, you can plan to be in it for 2 years. You will be asked to complete 4 study visits to collect data. These visits may occur in person, on Zoom (a video platform) or on the telephone. These visits are <u>not</u> part of any program session. Each data collection visit will have 2 parts: **Visit A** and **Visit B**. *Visit A* will take about 2 hours. This visit may

© 2025 Powell LH et al. *JAMA Intern Medicine*.

be in person or on a Zoom video call. *Visit B* will be in person and will take about 30 minutes. The first study visit is to see if you are able to be in the study. If you join the study, then you will return for study visits at 6 months, 15 months, and 24 months. The in-person visits will be held at Rush University Medical Center in Preventive Medicine. Research staff will call you every 3 months to ask you about your contact information and if you have been in the hospital or emergency room.

During these visits to collect data, you will complete a survey about your diet, stress and how active you are. We will measure your height, weight, size of your waist and your blood pressure. You will be given a lab order for a fasting blood sample. This means you cannot have any food or drink, except water for 12 hours before the blood sample is collected. You will wear a device around your waist for 7 days that will show how active you are. You will keep track of what you eat and make a plan for how you will be able to do the program.

After collecting the data, if you are still eligible to be in the study, you will be randomly assigned to one of two possible programs: the **Self-Directed Program** or the **Group-Based Program**. The *Self-Directed Program* requires you to read educational tip sheets and follow the instructions on your own; the tip sheets will be mailed/emailed to you. The *Group-Based Program* requires you to attend weekly group sessions for 3 months, biweekly group sessions for 3 months, and monthly sessions for 18 months. For a detailed list of study procedures, please see the "*Study Activities*" section of this consent form.

The risks associated with being in this study are low. There is a risk of loss of confidentiality if your medical information or your identity is obtained by someone other than study staff. We will take precautions to protect your identity and any medical information provided to us. All the information that is collected about you will be stored in a secure database. Your health information will be kept in a de-identified format. Only study staff will be given access to the portion of your data necessary to complete their job. Your identifying information will be deleted from the server ten years after the study is completed. Please do not share your medical information or social security numbers on any open forum or discussion boards.

Blood draw risk: You may feel pain or discomfort, and/or bleeding, and bruising at the site the needle enters the body, and in rare cases, fainting or infection as a result of blood draw. Blood will be drawn by a trained person and they will do everything to decrease any pain or discomfort you may feel.

Risk of having a reaction (allergy) to food served: Persons placed in the *Group-Based Program* will be asked to participate in cooking demonstrations. There will be a chance to taste many veggie dishes. If you have food allergies, you could have an allergic reaction to a food served during the session. To avoid having this reaction, we will ask you to list any foods you may react to or that you can't eat (such as dairy products, eggs, or other foods). All cooking classes will have a list of foods and items, such as spices, used in the veggie dish. You will be asked at each session if you may react or are allergic to any of the items in the recipe.

© 2025 Powell LH et al. *JAMA Intern Medicine*.

Risk of injury during physical activity: Persons placed in the *Group-Based Program* will be asked to do 20 minutes of movement or activity at the start of each session. You may feel some discomfort or get an injury while being active if you have not been active or moved a lot in a while. To lower this risk, you will be told to slowly start new activities, and to use warm-up and cool-down periods to lower the chance of getting hurt.

Benefit of Being in the Study

Being in this study may help you. Based on our use of these programs in people with similar conditions, the investigators think it may be helpful to people with your condition. However, no one can know if it will be helpful for you.

Other Options

There are other options to help you get healthy if you choose not to be in this study. You may choose another form of help or care for Mets without being in a study such as, joining a commercial weight loss or physical activity program. You may choose to do more physical activity, eat healthier foods, and lower your stress with the help of your doctor.

Detailed Information: Please review the rest of this document for details about the above topics and additional information you should know before making a decision about whether or not you will be in this study.

Why are you being invited to be in this study?

You are being asked to be in this study because you are at least 18 years of age. You are healthy with no history, symptoms, or signs of diabetes, heart or blood vessel disease or heart failure. Your tests suggest that you have metabolic syndrome. MetS includes at least three of the these: high blood sugar levels, high blood pressure, a large waistline, low levels of a healthy form of fat (HDL cholesterol), and high levels of a type of fat in the blood (triglycerides). MetS increases your risk of getting diabetes and heart failure. You have been asked to be in the study because you said you were interested in becoming healthier by making lifestyle changes and you are willing to test a lifestyle program.

How many participants will take part in this study?

About 600 participants are planned to take part in this study in the nation. There will be about 120 people from Rush.

Study Activities

Before you start the study

You will attend a 60-90 minute information session held on Zoom or in person. During this session, you will meet the study team and learn more about the programs and research study. If you still want to be in the study, we will plan your first study screening visit.

© 2025 Powell LH et al. *JAMA Intern Medicine*.

First Study Visit

Visit A

Your first study visit will be conducted in person or by a Zoom video (or by phone if you prefer) and will take about 2 hours. This visit is to see if you are able to be in the study. At this time, the study staff will go over the consent form and answer any questions you may have. We will then ask you some screening questions about your medical history and other info about you such as your age, ethnicity, and schooling. You will be asked many questions about your intake of fruits, veggies, and beverages; eating practices; health risk factors, habits, stress, alcohol, smoking and substance use, and other health- related questions. The study staff will review all of your current medications.

Home Assignment

After Visit A, you will be mailed a device to wear around your waist that will show us your activity level, a food log, and a sheet to make a plan for how you will do the ELM tasks each day. The activity device is hooked to a belt that you wear just above your right hip, and can be worn over or under your clothes. You may be asked to complete a written log to track your usage of this device. It takes less than 1 minute to put on and take off each day. We ask you to wear this device because it measures your total activity level and your step count each day. It does not measure the type of activity, where it is done, or anything else. **You will need to wear the activity device for 7 days in a row and at least 10 hours per day, write down the foods you eat in a food log, and fill out a short form about how you plan to eat more veggies and be more active, to be in the study. If you are not able to complete these tasks, you may not be able to be in the study.**

Visit B

If you meet the Visit A screening needs, you will make an appointment for about one week later to meet in person at Rush to complete Visit B. You will return the activity device when you come to this meeting. The research staff will check the device to make sure there are 7 complete days of recorded activity. You will also return the completed food log, and your plan for eating more veggies and doing more activity. At this visit, the research staff will note that you have done these tasks. **If you are not able to do the home tasks, you will have only one more chance to do it**. During this visit we will measure your weight, height, waist size and blood pressure. We will collect a blood sample (about 2 tablespoons) from a vein in your arm. You cannot eat or drink, except water for 12-hours before this lab test. From your blood tests, we will learn about your blood sugar levels, and hemoglobin A_{1C} (a test of long-term blood sugar control) and lipid levels (cholesterol).

You may not be able to be in the study: If the results from Visit B show that you do not have 3 or more risk factors for metabolic syndrome or you are not able to do the home tasks then you will not be able to be in the study. The research staff will let you know by phone or in person if

© 2025 Powell LH et al. *JAMA Intern Medicine*.

you are not able to take part in the study. We will then arrange for you to get your lab results and we will give you $20 as a thank you for your time.

How do I get placed into a program?

After we know that you can be in in the study, a computer will be used to place you in one of two programs: either the *Self-Directed Program* or the *Group-Based Program*. This means you are placed by chance, like a flip of a coin or roll of a dice. In the Self- Directed Program you will be given tip sheets on how to improve your lifestyle; these tip sheets will be mailed or emailed to you. In the *Group-Based Program* you will go to group sessions once a week for 3 months, then every other week for 3 months, and then once a month for 18 months. We have no control over what group you will be in. By signing this consent form you are agreeing to be in either program that the computer selects for you.

Lifestyle Programs

Self-Directed Program

If you are in the *Self-Directed Program*, you will be given:

- Tips to help you with MetS problems. These tip sheets were made by groups that focus on health problems such as MetS and have been tested and shown to help people like you. Tip sheets are sent every month by mail or email based on what you prefer. These will also be posted on the ELM website so you can go there to get all of the info during the study.
- A Fitbit, which is another type of activity device that you wear to track your activity. The Fitbit is yours to keep.
- Use of the ELM website that has all tip sheets and other info about the study.
- Progress letters with lab results that will be sent to you and, if you agree, to your doctor after each ELM study visit.

You will attend a 1-hour in-person session with the Self-Directed Coordinator. During this session, s/he will show you how to access the ELM website and find program materials. S/he will give you a Fitbit device and help you connect it to the Fitbit app. The Fitbit is yours to keep; you will not need to give it back at the end of the study.

Group-Based Program

If you are in the *Group-Based Program*, you will be given:

- A 1-hour in-person private session with a group leader (dietitian or health psychologist), once before starting group sessions and again 6 months into the program.
- Help with your MetS in a 90-minute group session. These group sessions are held once a week for 3 months, then every other week for 3 months, and then once a month for 18 months. For the first 6 months, sessions will include 20 minutes of some movement or activity, 20 minutes of nutrition info, and 50 minutes of talking as a group. In all, you will attend 37 group sessions over the 2-year period.

© 2025 Powell LH et al. *JAMA Intern Medicine*.

- A Fitbit, which is another type of activity device that you wear to track your activity. The Fitbit is yours to keep.
- A lifestyle log to track the food you eat and ELM Leaves for at least 6 weeks.
- Use of the ELM website that has other information about the study.
- Progress letters with lab results that will be sent to you and, if you agree, to your doctor after each ELM study visit.
- Use of an online community group using the Fitbit and ELM website to provide social support to other group members.

You will attend a 1-hour in-person session with a group leader. At this time, s/he will help you in making your own goals, give you a Fitbit, give you information about the group-based sessions, and explain how to use the ELM website.

As part of the *Group-Based Program* you will be given access to a web-based community group on Fitbit. An "ELM" group will be created for all to share their successes with others who are in the study. Being in the community group is your choice, provided for your help and its use is at your own risk. Rush does not control the content of the online program; has no control of what content is shown or not shown; does not have responsibility for any advice or information given and cannot promise the privacy of your data.

You will also be able to join an ELM community group on the ELM Website using a discussion board. This website is available to you without any promise of any kind. Any contact on the discussion board is at your own risk and any views, thoughts, and opinions expressed belong solely to the discussion board author.

The *Group-Based Program* sessions will be filmed or audiotaped or have pictures taken **only** for research and training purposes. These video or audio files or photos will be viewed by the research team to provide feedback to the group leaders at each site. The videos will be saved on a secure server **and will not be made public at any time.** The videos will be deleted from the server ten years after the study is completed.

Data Collection

All study participants (no matter what program you are in) will be asked to attend 4 study visits during the 2-year trial. This includes the first screening visit and then follow-up visits at 6, 15, and 24 months. The first screening visit and the visits at 6, 15, and 24 months require you to come to the study site at least once. Research staff will also call you every 3 months to review your contact info and ask if you have gone to the emergency room or been admitted to the hospital. Please see the table below that shows what will be done at the study visits.

© 2025 Powell LH et al. *JAMA Intern Medicine*.

Table of Study Activities

	In-Person/Zoom Contacts				Phone Contacts
	Baseline Visit A	Baseline Visit B	Months 6, 15, 24 Visit A	Months 6, 15, 24 Visit B	Months 3, 9, 12, 18, 21
obtain informed consent	x				
collect demographic, health, dietary and lifestyle info	x				
physical measurements*		x		x	
document medications		x		x	
fasting lab draw**		x		x	
questionnaires***	x		x		
dispense accelerometer	x		x		
participant to return accelerometer		x		x	
provide food log and logistical plan	x				
participant to return food log and logistical plan		x			
document adverse events			x		x
payment of participant stipend		x		x	

*Physical measurements to include:
weight, height, waist circumference, respiration rate and 3 blood pressure measurements

**Labs include:
Fasting blood glucose, lipids, hemoglobin A1c

***Questionnaires include:
NCI All Day Screener Fruits and Vegetables
Behavioral Risk Factor Surveillance System – Beverage Items
Eating Competence
Five Facet Mindfulness Questionnaire

© 2025 Powell LH et al. *JAMA Intern Medicine*.

Self-Report Habit Index
SF-36 Health Assessment
Social Support for Eating Habits Survey
Social Support and Exercise Survey
Perceived Stress Scale
Alcohol, Smoking and Substance Involvement Screening Test
Patient Health Questionnaire (PHQ-8)
Health Network Questionnaire

You will be given a Fitbit to track your activity and asked to wear it daily. You may have additional data collected. Data from your Fitbit may be transferred to Fitabase which is a tool that collects data from internet connected Fitbit devices allowing the study site and Research Coordinating Unit at Rush to see and analyze the data from your Fitbit. The purpose of the Fitabase data is to give you feedback on your activity to help you meet physical activity goals.

Additional Study Visit(s)
Additional study visits may be requested of you as a result of the COVID-19 pandemic effect on the study timeline. The ELM trial may decide to take a short pause to protect the health of participants and staff. If this occurs, the staff will inform you of when an additional visit can be expected. If the request is to complete the full set of study activities then you will be reimbursed $40 for your time. If the request is to complete a subset of activities, such as your blood work and physical measures then you will receive $20 for your time. You will also always receive the lab results from your study visits.

What do you need to know about the collection of blood samples?
In this study, we will collect a blood sample (an amount of about 2 tablespoons) from a vein in your arm. We will collect this as a fasting sample meaning that you will not have eaten, but had only water in the 12 hours before the test. This will occur at your first visit and at the 6, 15, and 24-month visits. These blood samples will be tested for blood sugar levels, hemoglobin A_{1C} (a test of your blood sugar over time) and lipid levels (cholesterol) by Quest Laboratories. The blood draw will occur at Rush's Department of Preventive Medicine Triangle Office Building, located at 1700 W. Van Buren Street, Suite 470.

Do you want your lab results sent to your medical provider?
If you agree, we will send your lab results to your doctor after your first visit at 6-months, 15-months, and 24-months. Please initial and date one of the following options:

_____ Initials	_________ Date	**Yes**, I agree to allow the ELM study to send my lab results to my medical provider.
_____ Initials	_________ Date	**No**, I do NOT agree to allow the ELM study to send my lab results to my medical provider

Will your information or blood samples be used for more studies later?

© 2025 Powell LH et al. *JAMA Intern Medicine*.

Information or blood samples collected from you for this study may be used for future research or shared with other researchers. If this happens, information that could show who you are will be removed before any information or blood samples are shared. Since there will be no information to let others know who you are, you will not be asked about letting us do this.

Can we contact you about being in future studies?
If you agree, we may contact you after this study about being in future studies. Please initial and date one of the following options:

________ ______________ Yes, I agree to be contacted about future research.
Initials Date

________ ______________ No, I do NOT agree to be contacted about future research.
Initials Date

New Study Findings
During this study, you will be told about important findings (either good or bad), such as changes in the risks of being in the study, how the study is helping you or new choices to being in the study that might cause you to change your mind about being in the study. If new info is shared with you, you may be asked to sign a new consent form to still be in this study.

Will you receive your individual results from the study?
In general, activities done for research purposes do not give clinical info. We may learn things about you from this study that could be useful to your health or treatment. The lab results (e.g. cholesterol, blood glucose, HbA1c) and physical measures (e.g. weight, height, blood pressure, waist circumference) collected at study visits will be shared with you. If you agree, we will also share your lab results with your doctor. We ask that you talk about these results with your doctor.

Can you leave or be removed from this study?
You have the right to leave this study at any time.

The study staff and sponsor also have the right to stop you from being in this study even if you want to stay in, if:

- They think it is in your best interests;
- You do not follow the study tasks and plan;
- The study is cancelled for any reason.

What about confidentiality of your medical information?
This authorization is voluntary. Rush University Medical Center and its affiliates ("Rush") will not withhold or refuse your treatment, payment, enrollment, or eligibility for benefits if you sign this authorization. You do not have to sign this authorization, but that means that you cannot be in the study or receive study-related treatment.

© 2025 Powell LH et al. *JAMA Intern Medicine*.

By signing this document, you voluntarily authorize (give permission to) Dr. Brad Appelhans, his study team, and other Rush personnel involved with the conduct and review of this study (which may include off-site personnel) to use or disclose (release) health information that identifies you for the study described in this document.

During the study, Dr. Brad Appelhans and his study team will collect Protected Health Information (PHI) about you for the purposes of this research. PHI is your health information that includes your medical history and new information obtained as a result of this study. Some of this information will come from your medical record. The health information that Rush may use or disclose for this research includes:

- Information from your medical record related to the metabolic syndrome, results of physical measures taken by research assistants, and lab tests conducted at 4 time points during the study.

Dr. Brad Appelhans and his study team may share your health information and the results of your study-related procedures and tests with people outside of Rush who assist with the conduct and review of this study. The persons who receive your health information may not be required by Federal privacy laws to protect it and may share your information with others without your permission, but only if permitted by the laws governing them. Your health information described above may be used or disclosed to:

- Collaborating Researchers (researchers that are performing the same study and are part of the study team) at the University of Colorado at Denver, the University of Missouri at Kansas City, Geisinger Clinic, and Rochester Institute of Technology;
- The study Sponsor, William G. McGowan Charitable Fund, and its representatives
- A Data Safety Monitoring Board composed of scientists who monitor study progress and safety;
- Rush University Medical Center, Department of Preventive Medicine;
- Monitoring agencies such as the Food and Drug Administration (FDA), the National Institutes of Health.

While you participate in the study you will have access to your medical record, but Dr. Brad Appelhans is not required to release to your study information that is not part of your medical record. Rush is required by law to protect your health information, and study records that identify you will be kept confidential. The results of study tests/procedures performed as part of this study may become part of your medical record. Any study information in your medical record will be kept indefinitely. Your identity will not be revealed on any report, publication, or at scientific meetings. All video tapes and pictures of participants obtained for research will be kept on a secure network for ten years after the study is completed. .

You have a right to inspect and copy the information to be disclosed with this authorization and you may obtain a copy of the information by contacting the office listed above.

© 2025 Powell LH et al. *JAMA Intern Medicine*.

If you no longer want to be in the study and do not want your future health information to be used, you may change your mind and revoke (take back) this authorization at any time by writing to Dr. Brad Appelhans at Department of Preventive Medicine, 1700 W. Van Buren, Suite 470, Chicago, Illinois 60612. If the authorization is revoked, you will no longer be allowed to participate in the study and previously authorized individuals/entities may still use or disclose health information that they have already obtained about you as necessary to maintain the integrity or reliability of the current study.

This authorization is valid for the entirety of this research study. It will expire upon your completion of the study or if you revoke (take back) the authorization.

If you withdraw from this study, the data already collected from you may not be removed from the study records. The study doctor and/or study team may ask you whether they can continue to collect follow-up data on you. If follow-up information will be requested, you will be asked to sign a separate consent form before this information can be collected.

Records of participation in this study will be maintained and kept confidential as required by law. All information collected from you will be de-identified using a random 8 digit number.

The Rush Institutional Review Board (IRB) will have access to your files as they pertain to this research study. The IRB is a special committee that reviews new and ongoing human research studies to check that the rules and regulations are followed regarding the protection of the rights and welfare of human participants.

A description of this clinical trial will be available on http://www.ClinicalTrials.gov, as required by U.S. Law. This Web site will not include information that can identify you. At most, the Web site will include a summary of the results. You can search this Web site at any time.

Certificate of Confidentiality

To help us protect you and the info we will be collecting from you, this study has gotten approval by the U.S. government. This means that researchers cannot be forced, even by courts or the police, to make known any info about you.

The does not stop you from making known, or agreeing in writing to allow study staff to make known, info about you. For example, if you would like an employer or insurer to know something about you that is documented in this study, you can write and sign a statement telling the study staff it is okay to give your employer or insurance company info.

For the protection of your privacy, study staff may not make known or use any information, documents, or specimens that could let others know who you are in any civil, criminal, administrative, legislative, or other legal way, unless you say it is okay. Info, documents, or specimens protected by this may be made known to someone who is not connected with the research:

© 2025 Powell LH et al. *JAMA Intern Medicine*.

(1) If there is a federal, state, or local law that requires release of information (such as to report child abuse or communicable diseases);
(2) if you consent to the release of info, including for your medical treatment;
(3) if it is used for other scientific research in a way that is allowed by the federal regulations that protect people in studies;
(4) for auditing or program evaluation by the government or funding agency if required by the United States.

This does not prevent you from choosing to release info about yourself. If you want your research info released to an insurer, medical care provider, or any other person not connected with the research, you must give permission to the study staff to release it. Please contact the lead study person for more info on how to provide this consent.

What are the costs to be in this study?
Being a part of this study will not mean added costs for you. However, you will need to pay for the costs of your usual medical care outside of the study, including doctor visits and any tests you may do.

Will you be paid for taking part in this study?
You will be paid $40 for completing the first, 6-month, and 15-month study visits. You will be paid $60 for the 24-month visit. If you are unable to participate in the study for any reason, you will be paid $20 for your time. If you do not finish this study, you will be paid for the study visits you have completed. You will be paid after completing the second meeting (Visit B) for each study visit. We may need to collect your social security number or Taxpayer Identification Number (TIN) in order to pay you and for tax reporting purposes to the United States Internal Revenue Service (IRS).

Being in this study may lead to new commercial products from which the Sponsor or others may gain a money benefit. No plans have been made to pay you for this.

What happens if I am injured or hurt during the study?
If you get ill or injured from being in the study, Rush University Medical Center will help you get medical treatment. You should let the study doctor know right away that you are sick or hurt. If you believe you have become ill or injured from this study, you should contact Dr. Brad Appelhans at telephone number (312) 942-3477.

You or your health insurance plan will be billed. No money has been set aside to pay the costs of this medical care. Health insurance plans may or may not cover costs of study-related injury or illness. You should check with your insurance company before deciding to be in this study. Costs not covered by insurance could be a lot.

Rush University Medical Center has no program to help you pay if you get hurt from being in this study. By signing this form, you are not giving up any legal rights to seek compensation of injury.

© 2025 Powell LH et al. *JAMA Intern Medicine*.

What other information should you know about?
Investigator Dual-Role
Your health care provider may be an investigator on this study, and as an investigator, is interested in both your clinical welfare and in the conduct of this study. Before entering this study or at any time during the study, you may ask for a second opinion about your care from a doctor who is not part of this study. You do not have to be in this study offered by your clinician. The decision to not participate will not affect your clinical care now or in the future.

Who can you contact for more information about this study?
Questions are encouraged. If you have further questions about this study, you may call Joselyn Williams, Project Director at (312) 563-8790 or email her at Joselyn_L_Williams@rush.edu.

Who can you contact if you have concerns about your rights as a study participant?
Questions about the rights of research participants may be addressed to the Rush University Medical Center Office of Research Affairs at 1-800-876-0772.

What are your rights as a study participant?
Taking part in this study is voluntary. If you choose not to participate in this study or to leave the study at any time, your health care, benefits or relationship at Rush University Medical Center will not change or be affected.

If you choose to leave this study and you do not want any of your information to be used, you must inform Dr. Brad Appelhans in writing at the address on the first page. Dr. Appelhans may still use your information that was collected prior to your written notice.

COVID-19/SARS-CoV-2 Pandemic Safety Precautions

We want to discuss the ways that we are protecting you and others during the COVID-19 SARS-CoV-2 pandemic. The COVID-19 virus is spread from person-to-person, and close contact with other people increases the risk of exposure to the virus. The risk of exposure is less by wearing a mask and staying at least 6 feet away from others whenever possible. The risk of contracting the virus is also lowered by excellent hand hygiene, which means frequent handwashing with soap. The ELM Trial is following recommendations from the State of Illinois, Cook County, the City of Chicago, and Rush University Medical Center to reduce the spread of COVID-19.

How WE plan to help keep all of us safe

In general, the following measures will be taken for any in-person activities as a precaution for COVID-19:

- Participants will be screened for COVID-19 symptoms on the phone or by text in advance of each in-person visit.
- Participants and staff will be screened for symptoms, and their temperature will be taken, immediately before any in-person meeting.

© 2025 Powell LH et al. *JAMA Intern Medicine*.

- Sanitizer or hand soap will be available and staff will encourage hand hygiene.
- Participants and staff will wear surgical masks provided by the trial at all times.
- Social distancing (6-foot separation) will be maintained with the exception of collecting physical measures.
- Participants will be encouraged to avoid touching their eyes, nose, or mouth.

How YOU can help keep all of us safe

For your safety and the safety of the staff, during ELM research activities, you agree to take precautions currently recommended by State and Local officials.

- You will attend an in-person meeting only if you are symptom free, such as no cough or fever.
- You will wash your hands or use alcohol-based hand sanitizer when you enter the building.
- You will follow the 6-foot social distancing precautions at all times, even when entering the building or using a rest room.
- You will always wear a mask over your mouth and nose.
- You will try not to touch your face, eyes, or mouth with your hands. If you do, you will immediately wash or sanitize your hands.
- You will take steps between meetings to minimize your exposure to COVID.
- If you, or a resident of your home, tests positive for the virus, you will immediately inform the research staff and, if possible, request a remote meeting.

Confidentiality

If someone in the ELM study tests positive for COVID-19, public health authorities may contact the study site for contact tracing. At that time, we would be required to confirm that the person had attended a study visit. We would also be required to name other participants and staff that the person had been in contact during the study visit.

If such a report is required, the minimum information necessary will be provided. No other health details will be provided about your ELM participation.

SIGNATURE BY THE PARTICIPANT:
I have read (or have had read to me) the contents of this consent form and have been told to ask questions. I have received answers to my questions. I give my consent to be in this study. I will receive a signed copy of this form for my records.

______________________	______________________	______________________
Name of Participant	Signature of Participant	Date of Signature

© 2025 Powell LH et al. *JAMA Intern Medicine*.

SIGNATURE BY THE INVESTIGATOR/INDIVIDUAL OBTAINING CONSENT:
I have read this form to the subject and/or the subject has read this form. An explanation of the research was given and questions from the subject were solicited and answered to the subject's satisfaction. In my judgment, the subject has demonstrated comprehension of the information.

____ ______________

Signature of Individual Obtaining Consent Date of Signature

SIGNATURE BY WITNESS/TRANSLATOR:
I observed the signing of this consent document and attest that, to the best of my knowledge, the person signing the consent form is the participant and the person signing the form has done so voluntarily.

____

Name of Witness/Translator

____ ______________

Signature of Witness/Translator Date of Signature

© 2025 Powell LH et al. *JAMA Intern Medicine*.

eMethods 3. Sensitivity Analyses

Pattern of Missing Data in ELM

In single-blind behavioral trials such as ELM, participants know which treatment they are receiving, treatment arms often vary in intensity and by time, and decisions to go missing are difficult to predict. Since there is no foolproof method for treatment of missing data, the ELM trial directed effort toward prevention of missing data over the long 2-year follow-up, resulting in an overall missing rate of 16.3%, less than the 20% missing assumed in the sample size calculations.

The pattern of missing data for the primary endpoint of metabolic syndrome (MetS) at 24 months was analyzed under the assumption that the data were *Missing Not at Random.* This was based upon observations that predictors of missingness at 6 months differed by Arm, and there were no measured predictors of missingness at 24 months. Specifically:

1. Differential Predictors of MetS Remission at 6 Months By Arm. Predictors of missing the 6-month exam differed by trial Arm. In the Treatment Arm, missingness was explained only by higher baseline body mass index (missing: 38.9; non-missing 35.9) and younger age (missing: 51.3 years vs. non-missing: 56.2 years). In the Comparator Arm, missingness was explained only by minority race (missing: Black=26.4%; White=12.6%).

2. No Predictors of MetS Remission at 24-Months. There were no significant baseline or 6-month predictors of missingness at the 24-month primary assessment in the total cohort or stratified by Arm. Of particular note, missingness was unrelated to 6-month improvement or lack of improvement. For those participants with 6-month data, neither MetS status, body mass index, nor weight at 6 months were related to missingness at 24 months. This argued against the common assumptions that participants become missing as a reaction to discouragement with lack of success, or satisfaction that goals have been reached and participation is no longer needed.

Analytic Strategies for Data *Missing Not at Random*

To prioritize protection of random assignment using intent-to-treat analyses, the strengths and limitations of the most common analytic approaches to missing data were drawn from general reviews,[1-3] critiques of common approaches,[4,5] special challenges in studies of weight management,[6,7] the emerging literature on *Missing Not at Random* approaches,[8] and the special case presented by binary outcome data.[9]

Multiple Imputation is a model-based imputation procedure that derives strength from taking uncertainty into account by using multiple plausible imputations.[1] But it was rejected because of the potential violation of the assumption of *missing at random*. If missingness cannot be explained by measured data, it provides a poor approximation for imputation of missing data leading to biased estimates.[10] Methods such as maximum likelihood were rejected for similar reasons.

Last Observation Carried Forward (LOCF) is a single imputation procedure that derives its strength from making use of all available data up until the time that a participant becomes missing. It is particularly well suited to trials of treatments showing a slope of early benefit followed by a plateau of sustainability over a long follow-up.[11] A limitation of this approach is the possibility that missingness results from non- or unsustained response to treatment.[5, 6] However, there was little evidence that missingness was related to response to treatment at 6 months, measured by change in MetS or weight. LOCF is also limited because it is a single imputation procedure that inflates certainty of result, leading to low estimates for standard errors.[1, 5] Thus, pairing it with an assessment of uncertainty in sensitivity analyses has been recommended.[8]

Pattern Mixture Modeling is appropriate for data that are *Missing Not at Random.*[8] It assumes that no approach to missingness is optimum and instead examines the robustness of a chosen primary analysis in the event of clinically plausible deviations from it.[8,12] Its limitation is that it depends upon clinical guesswork for clinically plausible deviations.

© 2025 Powell LH et al. *JAMA Intern Medicine*.

Scientific Justification: Last Observation Carried Forward with Pattern Mixture Modeling.
Little et al[1] cautioned against using LOCF without scientific justification. The scientific justification for using LOCF is five-fold.

1. LOCF makes use of actual measured data on the primary endpoint up until the time of missing. Actual measured status of MetS at 24 months was used in 517 (83.7%) of participants. MetS status at 24 months was imputed from actual measured data taken at 15 months in 9 (1.5%), at 6 months in 33 (5.3%), and at baseline in 59 (9.5%) with no difference in imputation rates between Arms.

2. LOCF does not require an assumption that missing data can be predicted by measured variables. This assumption cannot be supported since neither baseline nor 6-month measured data predicted missingness at the primary endpoint of MetS remission at 24 months in the total cohort or the cohort stratified by trial Arm.

3. LOCF works well when data plateau over a long follow-up. This is well aligned with the ELM trial that is designed as 2 distinct phases: initiation of MetS remission (0-6 months), and sustainability of MetS remission (6-24 months). The sustainability phase shows a MetS remission plateau with minimal change occurring from such things as delayed or unsustained improvement.

4. LOCF works well when missingness is not well explained by response to treatment. A common assumption in weight loss trials is that missingness is explained by a return to baseline,[5, 6] making LOCF an overly optimistic estimate of treatment response. This assumption is inconsistent with data showing that missingness at 24 months was unrelated to response to intensive treatment at 6 months.

5. LOCF single imputation has been paired with sensitivity analyses using pattern mixture models to provide an estimate of uncertainty. These sensitivity analyses show the impact of small deviations from LOCF imputed data on statistical significance.

Sensitivity Analyses
Table 1 presents statistics using LOCF imputation (MetS remission in Treatment and Comparator, percent difference in rates, adjusted Odds Ratios, 95% Confidence Intervals, and p-values). This is followed by sensitivity analyses for 6 tipping point scenarios that show the impact on statistical significance of clinically significant departures from the number of MetS remissions identified with LOCF. These scenarios progress from a loss of 1 or 2 MetS remissions in the Treatment arm with no change in Comparator, to a loss of 1 or 2 MetS remissions in the Comparator arm with no change in the Treatment Arm, to a loss of 1 or 2 MetS remissions in both Arms.

The tipping point analyses were carried out using pattern mixture models for binary data.[13, 14] Missing data were enumerated with MetS remission rates equivalent to the target tipping point scenarios in each arm. For example, in the scenario where 1 less MetS remission was assumed in the Treatment but no change in the Comparator (T= -1; C= 0 below), each missing value in the Treatment arm was imputed using a random Bernoulli draw such that the overall remission rate in the Treatment arm became approximately 27.5% (84/306), equivalent to one less remission compared to LOCF (85/306 = 27.8%). The Comparator arm was unchanged from LOCF in this scenario. This process was repeated 30 times for each scenario, and the results were averaged using Rubin's rule.

The results of the pattern mixture models show a range of adjusted odds ratios between 1.41 and 1.49. Small departures from LOCF, which could occur if a MetS remission at the most recent follow-up was not sustained at the final 24-month follow-up, reduce the statistical significance of the difference between arms. Across all departures, the percent difference between arms ranged between 27.8% and 35.6%. A percent difference of 25% between treatment arms was judged to be clinically meaningful and led to the early stopping of the SPRINT trial.[15,16] Statistical significance supported this percent difference in this trial given that it had 9,361 participants. By comparison, the ELM trial of 618 participants had significantly less power to detect a difference of this magnitude. The tipping point analyses in Table 1 can be considered conservative. First, they do not consider scenarios where the missingness may have led to increased overall remissions. Only scenarios where missingness decreased overall

© 2025 Powell LH et al. *JAMA Intern Medicine*.

remissions were considered. Second, 59 (58.4%) of the 101 LOCF imputations used the baseline value. Since all participants entered the trial with MetS, this is a conservative assumption that missing = a return to baseline for the majority of participants with missing 24-month MetS data.

Table 1. Sensitivity Analyses

	**TREATMENT** **# MetS Remissions/ at risk (%)**	**COMPARATOR** **# MetS Remissions/ at risk (%)**	**Percent Difference in Rates**	**Adusted Odds Ratio[a]**	**95% Confidence Interval**	**p-value**
**Last Observation Carried Forward**	**85/306 (27.8%)**	**66/312 (21.2%)**	**31.1%**	**1.46**	**1.01-2.14**	**0.049**
**Pattern Mix: Tipping Points[b]** Change in #Events from LOCF						
T= -1; C= 0	84/306 (27.5%)	66/312 (21.2%)	29.7%	1.41	0.95-2.07	0.085
T= -2; C= 0	83/306 (27.1%)	66/312 (21.2%)	27.8%	1.41	0.96-2.07	0.084
T= 0; C= -1	85/306 (27.8%)	65/312 (20.8%)	33.7%	1.46	0.99-2.17	0.059
T= 0; C= -2	85/306 (27.8%)	64/312 (20.5%)	35.6%	1.49	1.00-2.22	0.051
T= -1; C= -1	84/306 (27.5%)	65/312 (20.8%)	32.2%	1.45	0.98-2.16	0.063
T= -2; C= -2	83/306 (27.1%)	64/312 (20.5%)	32.2%	1.46	0.99-2.16	0.058

References

© 2025 Powell LH et al. *JAMA Intern Medicine*.

[1]Little RJ, D'Agostino R, Cohen ML et al. The prevention and treatment of missing data in clinical trials. *NEJM* 2012;367:1355-1360. doi: 10.1056/NEJMsr1203730.

[2]Molenberghs G, Kenward MG. *Missing Data in Clinical Studies*. Chicester:Wiley; 2014. doi.10.1002/9780470510445.

[3]Enders CK. Analyzing longitudinal data with missing values. *Rehab Psych* 2011;56:267-288. doi: 10.1037/a0025579.

[4]Davis MP. Missing data and last observation carried forward. *J Pain Symp Mgmt* 2024;67:921-922. doi.org/10.1016/j.jpainsymman.2024.02.019.

[5]Lachin JM. Fallacies of last observation carried forward. *Clin Trials* 2016;13:161-168. doi: 10.1177/1740774515602688.

[6]Ware JH. Interpreting incomplete data in studies of diet and weight loss. *NEJM* 2003;348: 2136-2137. doi: 10.1056/NEJMe030054.

[7]McEvoy BW. Missing data in clinical trials for weight management. *J Biopharm Stat* 2016;26:30-36. doi.org/10.1080/10543406.2015.1094814.

[8]Leurent B, Gomes M, Faria R, Morris S, Grieve R, Carpenter JR. Sensitivity analysis for not-at-random missing data in trial-based cost-effectiveness analysis: A tutorial. *PharmacoEcon* 2018;36:889-901. doi.org/10/1007/s40273-018-0650-5.

[9]Jackson D, White IR, Mason D, Sutton S. A general method for handling missing binary outcome data in randomized controlled trials. *Addiction* 2014;109:1986-1993. doi: 10.1111/add.12721.

[10]Hughes RA, Heron J, Sterne JAC, Tillian K. Accounting for missing data in statistical analyses: Multiple imputation is not always the answer. *Intl J Epidemiol* 2019;48:1294-1304. doi: 10.1093?ije/dyz032.

[11]Bourguignon L, Lukas LP, Guest JD, et al. Studying missingness in spinal cord injury data: Challenges and impact of data imputation. *BMC Med Res Meth* 2024;24:5. doi.org/10.1186/s12874-023-02125-x.

[12]Ratitch B, O'Kelly M, Tosiello R. Missing data in clinical trials: from clinical assumptions to statistical analysis using pattern mixture models. *Phar Stat* 2013;12:337-347. doi: 10.1002/pst.1549.

[13]Bunouf P, Molenbergs G. Implementation of pattern-mixture models in randomized clinical trials. *Pharm Stat.* 2016;15:494-506. doi: 10.1002/pst.1780. Epub 2016 Sep 23.

[14]Sui Y, Bu X, Duan Y, Li Y, Wang X. Application of tipping point analysis in clinical trials using the multiple imputation procedure in SAS .*Pharmasug.org/PharmSUG*-2023-SD-069.

[15]The SPRINT Research Group. A randomized trial of intensive versus standard blood-pressure control. *NEJM* 2015;373:2103-2116. doi: 10.1056/NEJMoa1511939.

[16]The SPRINT Research Group. Final report of a trial of intensive versus standard blood-pressure control. *NEJM* 2021:384:1921-1930. doi: 10.1056/NEJMoa1901281.

eMethods 4. Data Sharing and Dissemination

DATA AVAILABLE: No

© 2025 Powell LH et al. *JAMA Intern Medicine*.

ADDITIONAL INFORMATION

Explanation for availability of data

- The de-identified individual participant data, corresponding data dictionary, and analytic code will not be made publicly available. However, the data may be made available to qualified researchers upon reasonable request through a formal proposal to the Principal Investigator (Lynda Powell, PhD), contingent upon approval by the Institutional Review Board (IRB) and execution of a data use agreement.
- **What may be shared:** De-identified individual-level data and a data dictionary.
- **When:** Data may be available beginning 12 months after publication and up to 3 years post-publication.
- **Where/how:** Upon request and subject to institutional data sharing procedures.
- **Permitted analyses:** Requests must include a methodologically sound proposal for research consistent with the original study objectives.
- **Restrictions:** Data access is subject to IRB approval and a formal data use agreement. Statistical code and supporting documentation will not be shared.

© 2025 Powell LH et al. *JAMA Intern Medicine*.

eMethods 5. Table 2 (Addition)

Table 2 (Addition). Secondary outcomes at 6 and 24 months: Effect sizes and 95% confidence intervals[a]

	6 Months		24 Months	
	**Estimate**	**95% CI**	**Estimate**	**95% CI**
**CLINICAL**				
Metabolic Syndrome Remission	0.496	0.064, 0.928	0.381	0.0001, 0.761
Waist, mean (SD), cm	-2.758	-4.096, -1.420	-0.983	-2.396, 0.431
Systolic Blood Pressure, mmHg	0.957	-1.314, 3.227	1.213	-1.129, 3.555
Diastolic Blood Pressure, mmHg	0.187	-1.113, 1.488	-0.046	-1.645, 1.552
Triglycerides, mg/dL	-12.114	-21.052, -3.176	-3.777	-15.151, 7.597
Fasting Glucose, mg/dL	-2.335	-4.138, -0.533	-2.735	-5.309, -0.161
HDL Cholesterol, mg/dL	0.895	-0.281, 2.071	0.543	-0.738, 1.824
Body Mass Index	-1.124	-1.529, -0.718	-0.340	-0.866, 0.187
Weight, kg	-3.068	-4.188, -1.948	-0.919	-2.405, 0.567
LDL Cholesterol, mg/dL	-1.550	-5.146, 2.046	-2.442	-6.573, 1.689
HgA1c, %	-0.091	-0.166, -0.017	-0.063	-0.152, 0.026
Metabolic Syndrome Severity	-0.157	-0.235, -0.078	-0.092	-0.187, 0.003
Quality of Life				
Perceived Health	4.114	2.119, 6.108	1.749	-0.535, 4.033
Energy/Vitality	5.703	3.054, 8.352	2.554	-0.660, 5.768
Depressive Symptoms	-0.470	-0.973, 0.033	0.096	-0.423, 0.615
Perceived Stress	-1.253	-2.123, -0.384	-0.455	-1.447, 0.538
**LIFESTYLE TARGETS OF TREATMENT**				
Vegetable Intake, servings/day	1.143	0.712, 1.575	0.515	0.038, 0.991
Moderate-Intensity Physical Activity, min/week	18.350	3.541, 33.159	14.417	-6.843, 35.677
Daily Steps	727.308	370.709, 1083.906	432.511	66.438, 798.583
Sensory Awareness	0.223	0.140, 0.307	0.169	0.080, 0.259
Emotion Regulation	-0.006	-0.079, 0.067	0.030	-0.053, 0.113
Automatic Habits				
Brisk walks	0.358	0.227, 0.490	0.272	0.122, 0.422
Vegetables at meals	0.186	0.059, 0.312	0.105	-0.026, 0.235
Notice sensory experiences	0.064	-0.059, 0.187	0.082	-0.044, 0.208
Pause before reacting	0.089	-0.023, 0.201	-0.005	-0.125, 0.116

[a]Adjusted for clustering and the prespecified baseline covariates of age, sex, race/ethnicity, education, income, geographic site, body mass index, and number of co-morbidities.

© 2025 Powell LH et al. *JAMA Intern Medicine*.

eFigure 1. Metabolic syndrome remission by trial arm and time, including the 15-month assessment[a]

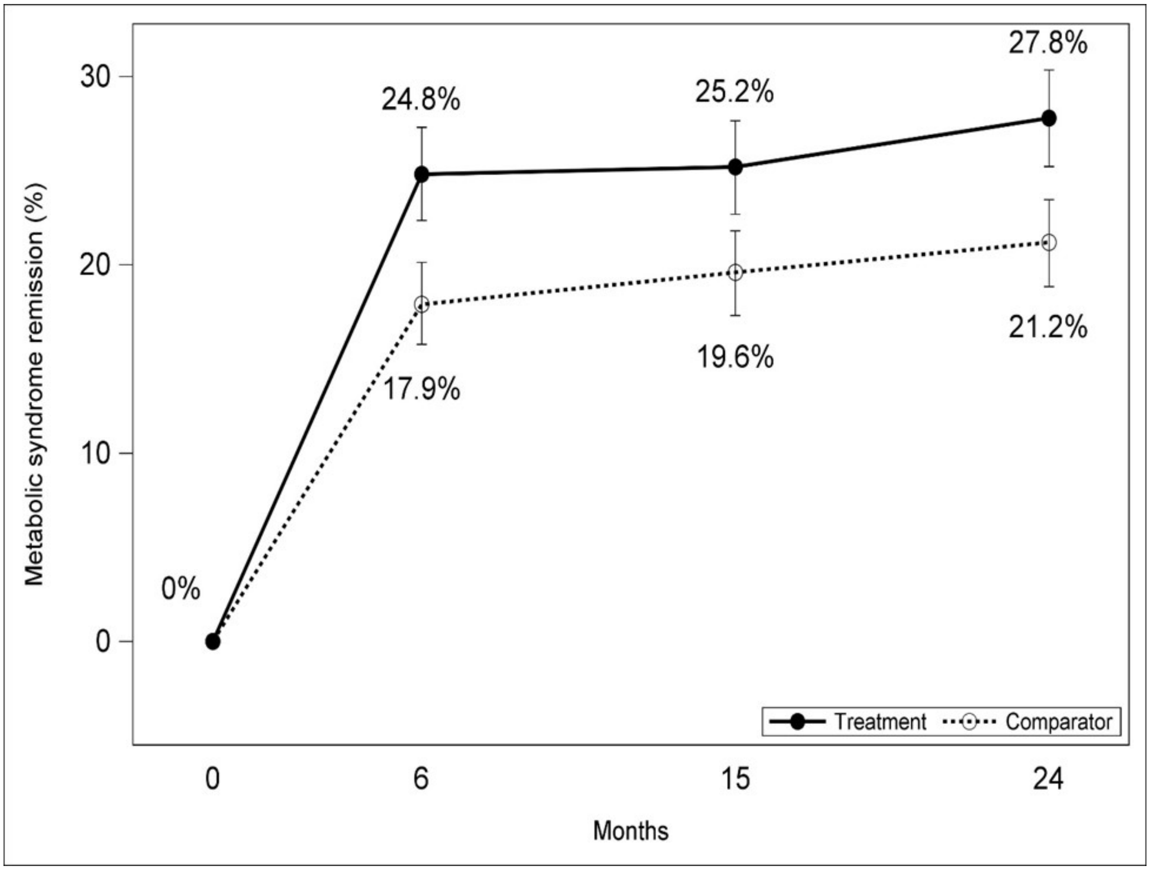

[a] Data on metabolic syndrome remission at 15 months using last observation carried forward are presented here for completeness only. They are not presented as the primary outcome because: (1) trial hypotheses focus only on two time points: initiation at 6 months and sustainability at 24 months; (2) the 15-month assessment was primarily a retention strategy; (3) COVID-19 resulted in compression of time for assessing the primary endpoint, overlap in timing of the 15- and 24-month assessments, and the priority given to the 24-month assessment; and (4) the resulting amount of missing data at 15 months was unacceptably high (22.5% missing); higher than missing at 6 months (14.5%) and 24 months (16.3%), and higher than the target rate in the sample size calculations of $\leq$20%.

© 2025 Powell LH et al. *JAMA Intern Medicine*.

eFigure 2. Prespecified baseline subgroups: Odds of MetS remission at 6 and 24 months

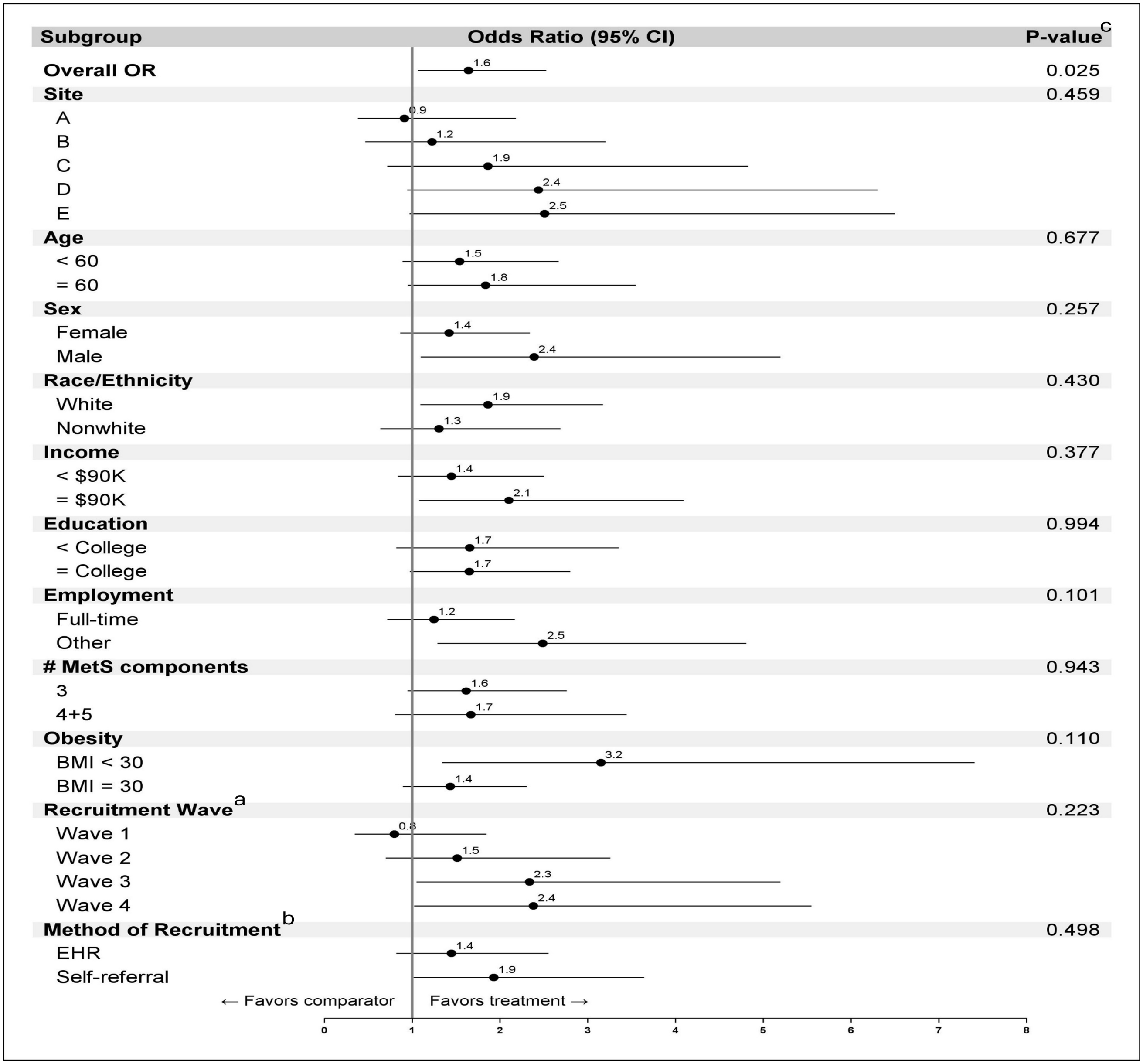

[a]Recruitment was conducted in 4 successive waves of appropriately 150/wave.
[b]Self-referral was primarily response to social or mass media advertisements and included a small number of other miscellaneous methods.
[c]Overall OR p-value is for the overall cohort. OR for subgroups is the p-value for the interaction term (treatment x subgroup) from a mixed model with adjustment for treatment, subgroup, cluster, and prespecified covariates.

© 2025 Powell LH et al. *JAMA Intern Medicine*.

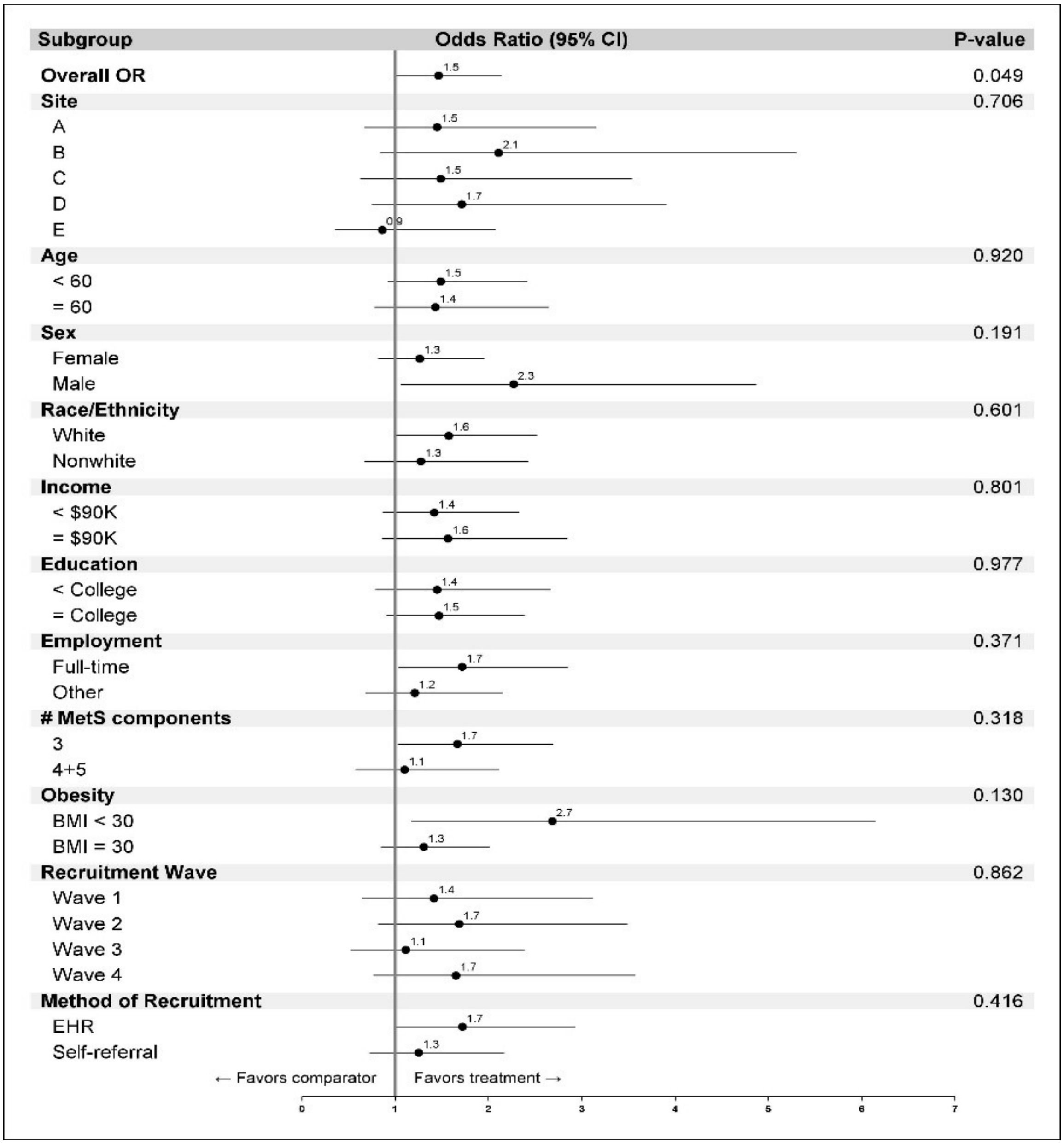

[a]Recruitment was conducted in 4 successive waves of appropriately 150/wave.
[b]Self-referral was primarily response to social or mass media advertisements and included a small number of other miscellaneous methods.
[c]Overall OR p-value is for the overall cohort. OR for subgroups is the p-value for the interaction term (treatment x subgroup) from a mixed model with adjustment for treatment, subgroup, cluster, and prespecified covariates

© 2025 Powell LH et al. *JAMA Intern Medicine*.
