# Supplementary material for: Lifestyle Intervention for Sustained Remission of Metabolic Syndrome: A Randomized Clinical Trial
Source: JAMA Intern Med. 2025 Nov 9;186(1):67–77. doi: 10.1001/jamainternmed.2025.5900 (PMC12598583; doi:10.1001/jamainternmed.2025.5900)
Supplement: Supplement 3. — Nonauthor Collaborators [file jamainternmed-e255900-s003.pdf]

Supplemental Online Content: Nonauthor Collaborators
*First name, last name, and suffix (if applicable) are required and will appear in PubMed.

*Group Name(s): ELM Trial Research Group							
***First Name and Middle Initial(s)**	***Last Name**	***Suffix (eg, Jr, III)**	Academic Degrees	Institution	Location (city, state/province, country)	Role or Contribution, eg, chair, principal investigator	Group (if more than 1 Group listed in the byline) and/or Subgroup (eg, Steering Committee)
Robert M	Kaplan		PhD	Stanford University	Stanford, CA	Chair	Data and Safety Monitoring Board
Walter T	Ambrosius		PhD	Wake Forest University	Winston-Salem, NC	Member	Data and Safety Monitoring Board
Emily	Anderson		PhD	Loyola University	Chicago, IL	Member	Data and Safety Monitoring Board
Cora	Lewis		MD	University of Alabama-Birminghan	Birmingham, AL	Member	Data and Safety Monitoring Board
Gerri	Norington				Chicago, IL	Member	Data and Safety Monitoring Board
Joyce	Mack			Rush University Medical Center	Chicago, IL	Administrative Assistance	
Elise M	Winn		BS	Rush Medical College	Chicago, IL	Editing Assistance	
Paul D	Glover		BS	Rush University Medical Center	Chicago, IL	Data Coordinator	Data Coordinating Center
Daniel R	Lindich		BS	Northwestern University	Chicago, IL	Project Director	Data Coordinating Center
Barbara	Mascitti		BS, RDN	Rush University Medical Center	Chicago, IL	Project Manager	Data Coordinating Center
Tami	Olinger		MS	Rush University Medical Center	Chicago, IL	Data Manager	Data Coordinating Center
Heidi E	Schroeder		BS, RDN	California Polytechnic State University	San Louis Obispo, CA	Director	Intervention Coordinating Unit
Bryce T	Daniels		PhD	University of Arkansas	Fayetteville, AR	Co-Director	Intervention Coordinating Unit
Jeanne Anne	Breen		MS	University of Colorado-Denver	Denver, CO	Project Coordinator	Denver Clinical Site
April	Hamilton		BS	University of Colorado-Denver	Denver, CO	Project Coordinator	Denver Clinical Site

Supplemental Online Content: Nonauthor Collaborators
*First name, last name, and suffix (if applicable) are required and will appear in PubMed.

***First Name and Middle Initial(s)**	***Last Name**	***Suffix (eg, Jr, III)**	Academic Degrees	Institution	Location (city, state/province, country)	Role or Contribution, eg, chair, principal investigator	Group (if more than 1 Group listed in the byline) and/or Subgroup (eg, Steering Committee)
Lisa	Bailey-Davis		DEd, RD	Geisinger	Central/Northeast, PA	Site Principal Investigator	Central/Northeast Clinical Site
Jamie	Loughney		BS	Geisinger	Central/Northeast, PA	Project Coordinator	Central/Northeast Clinical Site
Jacob	Mowery		BA	Geisinger	Central/Northeast, PA	Project Coordinator	Central/Northeast Clinical Site
Jenna	O'Donnell		BS	Geisinger	Central/Northeast, PA	Project Coordinator	Central/Northeast Clinical Site
Pavani	Chilamkuri		MBBS, MPH	University of Missouri-Kansas City	Kansas City, MO	Data Collection	Kansas City Clinical Site
Bong	Nguyen		PhD	University of Missouri-Kansas City	Kansas City, MO	Project Coordinator	Kansas City Clinical Site
Mariah	Pratt		BS	University of Missouri-Kansas City	Kansas City, MO	Data Collection	Kansas City Clinical Site
Kristie	O'Connor		MLA, RDN	Rochester Institute of Technology	Rochester, NY	Project Coordinator	Rochester Clinical Site
Dan	Ornt		MD	Rochester Institute of Technology	Rochester, NY	Site Co-Principal Investigator	Rochester Clinical Site
Catherine	Feit		BSN	Rush University Medical Center	Chicago, IL	Data Collection	Chicago Clinical Site
Serina	Silvestry		BS	Rush University Medical Center	Chicago, IL	Data Collection	Chicago Clinical Site
