# Supplementary material for: Lifestyle Intervention for Sustained Remission of Metabolic Syndrome: A Randomized Clinical Trial
Source: JAMA Intern Med. 2025 Nov 9;186(1):67–77. doi: 10.1001/jamainternmed.2025.5900 (PMC12598583; doi:10.1001/jamainternmed.2025.5900)
Supplement: Supplement 4. — Data Sharing Statement [file jamainternmed-e255900-s004.pdf]

Data Sharing Statement

Powell. Lifestyle Intervention for Sustained Remission of Metabolic Syndrome. *JAMA Intern Med*. Published November 09, 2025. doi:10.1001/jamainternmed.2025.5900

Data
Additional Information: clinicaltrials.gov: NCT04036006
Data available: Yes
Data types: Deidentified participant data, Data dictionary
How to access data: Request for data must be sent to the Principal Investigator, Dr. Powell, lpowell@rush.edu
When available: With publication

Supporting Documents
Document types: Statistical/analytic code, Informed consent form
How to access documents: Informed Consent form is presented in online eMethods 2. Statistical/analytic code will be available upon request to the Principal Investigator, Dr. Powell, at lpowell@rush.edu.
When available: With publication

Additional Information
Who can access the data: Researchers whose proposed use of the data has been approved.
Types of analyses: For a specified purpose.
Mechanisms of data availability: With or without investigator support, after approval of a proposal, and with a signed data access agreement.
Any additional restrictions: None
